# A feature-optimized EEG framework using multiscale entropy and metaheuristic optimization for cognitive analysis

**DOI:** 10.3389/frai.2026.1888234

**Published:** 2026-10-05

**Authors:** Keerthika N., Kiruthika V.

**Affiliations:** School of Electronics Engineering, Vellore Institute of Technology Chennai, Chennai, Tamil Nadu, India

**Keywords:** cognitive analysis, EEG, feature selection, machine learning, multiscale feature extraction

## Abstract

**Background:**

Cognitive activity plays an important role by influencing working-memory, problem-solving, attention, response and decision making. EEG serves as a non-invasive approach to analyze neural activity associated with cognitive processing. The current study aims to identify the more contributing features to analyze the cognitive activity between sports and non-sports groups.

**Methods:**

The present study included 100 participants, consisting of 80 males and 20 females aged between 19 and 23 years. EEG signals were acquired and preprocessed using filtering methods to remove unwanted noise and artifacts. The four-entropy metrics dispersion entropy, permutation entropy, sample entropy, and fuzzy entropy were applied to the coarse-graining method across multiple temporal scales to analyze the complexity of EEG signals. To identify the most informative feature subsets and reduce redundancy, filter-based methods such as Mutual Information, Pearson Correlation, Minimum Redundancy Maximum Relevance and wrapper-based methods such as Genetic Algorithm and Particle Swarm Optimization were applied. Finally for differentiating the cognitive activity, five machine learning classifiers that include Support Vector Machine, Artificial Neural Network, *K*-nearest neighbor, Decision Tree and Random Forest were applied. Statistical methods such as *T*-test and Fleiss kappa were used to analyse the performance efficiency.

**Results:**

Feature extraction phase yielded a total of 160 multiscale entropy features. The entire features when fed into both filter-based and wrapper-based methods, Genetic Algorithm based optimization yielded a minimal contributing feature subset with high nested cross-validated accuracy of 93.0 ± 2.7% with Support Vector Machine. This contributing feature subset was used as input for five different machine learning classifiers for differentiating the cognitive activity between both groups. The five classifiers demonstrated high cross-validated agreement on held-out subjects, with a mean Fleiss' kappa of 0.874 ± 0.051 (95% CI: 0.811–0.937), depicting a good inter-classifier agreement for classification of samples into sports and non-sports individuals.

**Discussion:**

The study demonstrates that multiscale entropy features combined with metaheuristic-based feature optimization can effectively analyze the cognitive activity between sports and non-sports individuals. The proposed EEG framework provides a structured approach for real-time cognitive activity analysis using compact and informative entropy-based feature subsets.

## 1 Introduction

Physical activities play an important role in cognitive enhancement by means of integrated mind-body interactions. When compared to aerobic exercises or resistance training, more movements are required in activities associated with sports, which intensifies cortical activity in the brain. Exercise improves blood flow in the brain, boosting fitness and cognitive functions (Douris et al., 2015). It has been stated that sport activities are characterized by improved behavior performance with unique brain activity when compared to non-sport activities (Molcho et al., 2022; Komarudin et al., 2022). The connection between physical fitness test and cognitive achievement is particularly applicable in competitive-based sports like soccer, gymnastics and basketball where victory occasionally depends on prompt decision-making process and agility (Vaughan and Laborde, 2021; Keerthika and Kiruthika, 2024). Competitive play requires professional sportsmen to process numerous actions that are dissimilar at a time. In order to measure cognitive activity, there are several methodologies used, such as the Stroop Test, the Wechsler Digit Spell Forward and Backward Test, and the Trail Making Test, for monitoring gaze attentions and visual attention (Onate et al., 2000). These tests give information about the situational awareness evaluation and decision-making process in a sports framework. However, such tests are normally conducted under controlled conditions, which may not capture the complexities and dynamics of a real-world situation (Nicolas-Alonso and Gomez-Gil, 2012). Electroencephalography (EEG) enables a real-time analysis of the brain activity. Cognitive activity relies on brain network activity, in which numerous neurons that interrelate with one another help to facilitate mental processes and sustain important physiological processes. EEG is a non-invasive method of measuring that follows the 10–20 International electrode placement (Klem et al., 1999). EEG has been utilized to investigate brain activity in relation to sport-specific demands. A study examined the 32-channel wireless EEG to compare cortical activity between elite athletes and non-athletes in basketball free-throw and pass-catching tasks. The results have shown more pronounced cortical activation in elite athletes in all EEG frequency bands, as determined by means of spectral power density analysis (Keshvari et al., 2025). In another study, mobile EEG has been recorded in the process of football-kicking exercise to evaluate the brain complexity in terms of expert knowledge; non-linear features computed at 64 temporal scales in frontal and centroparietal brain regions showed differences between football players and novices along with the link between entropy and kicking accuracy (Piskin et al., 2026). 16-channel EEG was applied for the determination of differences between cognitive and resting conditions by means of statistical EEG features obtained in the process of mental arithmetic task (Karnan et al., 2025). Similarly, six EEG locations have been chosen to analyze cognitive processes using neurofeedback training (Maaz et al., 2026). Cognitive activity pertaining to attention, working memory, and decision-making is mostly present in the frontal and parietal regions, especially in F3 (Valdés-Alemán et al., 2025), F4 (Misselhorn et al., 2019), P3 (Swerdloff and Hargrove, 2023), and P4 (Jiang, 2005) electrode positions. The frequencies contributing to cognitive analysis are found in alpha and beta (8–13 Hz and 14−30 Hz, respectively) (Keerthika and Kiruthika, 2024; Keerthika et al., 2024, 2025). Hence, the present study aims to develop an EEG-based methodology for identifying feature-level differences in cognitive activity between sports and non-sports groups during a mental-arithmetic task.

## 2 Related study

### 2.1 EEG preprocessing and feature extraction

Preprocessing is vital in enhancing signal quality by reducing noise and artifacts without eliminating task relevant neural activity. Various filters were used (low-pass, high-pass, band-pass and band-stop) to cancel baseline drift, high frequency components, and undesired frequency content outside cognitive band of interest (Keerthika and Kiruthika, 2024; Onate et al., 2000; Dai et al., 2019). Simple filtering cannot be done when artifacts are of the same frequency as actual EEG activity. In line with this, the blind source separation methods are used to break down the multichannel EEG into independent or orthogonal components using Independent Component Analysis (ICA) and Principal Component Analysis (PCA). This breakdown decomposes the EEG into components in which artifacts like eye blinking, muscle activity and other physiological noise occur as separate components (Delorme et al., 2007; de Cheveigné and Simon, 2007). In addition, Empirical Mode Decomposition (EMD) and Variational Mode Decomposition (VMD) can be used to obtain a non-stationary component of the EEG signals by decomposing the signal to intrinsic mode functions or variational modes. These methods will eliminate the noise elements and leave the cognitive content of the EEG signal (Sukriti and Mitra, 2020, 2021a). A study investigated background-vs.-arithmetic-state classification using VMD and adaptive boosting algorithm which gave highest classification accuracy of 96% (Saini et al., 2020). Filtering and ICA have been employed as EEG preprocessing procedures to reduce noise and artifacts before analyzing mental workload and mental-arithmetic activity (Qu et al., 2020; Zyma et al., 2019). These studies support the complementary use of filtering and ICA to improve EEG signal quality before cognitive-activity analysis. Accordingly, these preprocessing techniques were applied in the present study before feature extraction and classification. This method involves the analysis of EEG segments in the time, frequency and time-frequency domain to extract relevant information (Jiang, 2005). To better understand and study how the brain works, several studies have used a number of linear and nonlinear features (Keerthika et al., 2025; Keerthika and Kiruthika, 2024; Dai et al., 2019; Delorme et al., 2007). The most common linear EEG features generally include time domain features such as mean, absolute mean, variance, standard deviation, skewness, root mean square, kurtosis, and autocorrelation. Hjorth activity, mobility, and complexity peak-to-peak amplitude are also frequently used as time-domain descriptors. Frequency-domain features generally include absolute and relative power in the delta, theta, alpha, beta, and gamma bands, total spectral power, peak frequency, mean frequency, and band-power ratios. These linear features have been generally used in EEG analysis (Singh and Krishnan, 2023; Al-Fahoum and Al-Fraihat, 2014; Hjorth, 1970). Nonlinear EEG features such as entropy, fractal, and complexity-based measures are used to characterize the irregular and dynamic behavior of brain signals. Frequently extracted features include approximate entropy (ApEnt), sample entropy (SaEnt), fuzzy entropy (FzEnt), permutation entropy (PrEnt), dispersion entropy (DpEnt), Shannon entropy (ShEnt), spectral entropy (SpEnt), Higuchi and Petrosian fractal dimensions, and Lempel–Ziv complexity. Multiscale extensions of entropy measures are also applied to examine EEG complexity over multiple temporal scales (Keerthika et al., 2025, 2024).

The entropy value is usually lower for regular and predictable signals and higher for irregular and complex signals. Therefore, entropy is a useful measure for the analysis of cognitive activity. SaEnt measures the predictability of EEG patterns at a given scale, while FzEnt improves robustness by using a fuzzy similarity function instead of a hard threshold. PrEnt and DpEnt further describe complexity from ordinal/dispersion pattern distributions, providing efficient markers of temporal structure across scales (Sukriti and Mitra, 2020, 2021a). PrEnt was used to analyze the Mild Cognitive Impairment to quantify changes in EEG signal and detect the early cognitive problems (Fernández-Valdés et al., 2023). A study analyzed movement variability during tackle drills using SaEnt. It reduced variability across successive tackle blocks for the rugby players (Munia et al., 2017). Another study analyzed mild amnesia and memory loss for the soccer players using approximate entropy (ApEnt), to evaluate differences in brain activity and cognitive function (Eroglu et al., 2020). Multiscale Sample Entropy (MSaEnt) was used to analyze the cognitive function in older adults (Nawaz et al., 2022). Another study conducted an in-depth analysis of SaEnt, FzEnt, and PrEnt in multiple scales to identify the epileptic seizures (Sukriti and Mitra, 2021a). A study examined the EEG signals recorded during prolonged music listening to identify emotional responses. Signal complexity was measured using ApEnt and SaEnt, and the K-nearest neighbor (KNN) classifier achieved an accuracy of over 80% (Lu et al., 2022).

### 2.2 Feature selection and optimization methods

Traditionally feature-selection techniques can be categorized into various methods, namely filter, wrapper and embedded- based methods (Xu et al., 2023). To compare the dependence of linear and non-linear variables on cognitive activity, Mutual Information (MI) was used to explore the dependence of two variables using mutual and marginal distributions of the shared information (Arslan, 2018). The Minimum Redundancy Maximum Relevance (mRMR) method selects features with minimizing the redundancy and maximizing relevant features with the target class, thereby reducing repeated information. This approach has been widely used in pattern classification problems because it improves feature selection efficiency and supports better classification accuracy (Peng et al., 2005; Al-Nafjan, 2022). Pearson correlation method measures the linear association between each EEG feature and the target variable or class label. Features with higher absolute correlation values are considered more relevant because they show stronger separability between classes (Pawan, 2023). The Genetic Algorithm (Ga) was explored in an EEG study to get a combination of feature subset along with their contributing electrode positions to screen cognition. Sub-band representations were derived and a sparse set of channel features was chosen, which retained subject-wise generalizability, indicating a higher level of separability that could be achieved with minimal features (Aljalal et al., 2024; Houssein et al., 2024). The cognitive state detection through EEG was investigated by a wrapper-type feature-selection model: Particle Swarm Optimization (PsO) was used to find a compact and discriminative subset within a large set of features with accuracy of 90.25% using KNN (Firpi and Vogelstein, 2011; Lahiri et al., 2017).

### 2.3 Research gap, contributions, and challenges

Most of the EEG signals used for analysis in sports applications rely mainly on the simple linear and non-linear features, with non-linear methods usually restricted to the single-scale entropy measures (Tang and Li, 2024; Wan et al., 2024). From the above literature survey, the following gaps are identified

- Very few studies have explored the impact of cognitive activity in sports using EEG.
- Advanced non-linear and multiscale entropy (MEnt) features are still underexplored.
- Extensive investigation on feature level contribution in cognitive analysis is not fully explored.

To overcome these limitations, the proposed study develops an EEG based feature analysis framework for analyzing the cognitive activity with the following contributions.

- To extract multiscale entropy features from EEG signal.
- To analyze the impact of extracted features using advanced filter and wrapper-based techniques.
- To identify the more contributing features for cognitive activity analysis.
- To differentiate and compare the cognitive activity between sports and non-sports individuals.
- To validate the developed framework on a benchmark dataset.

To achieve the above-mentioned contributions, the following challenges were encountered.

- EEG acquisition using the OpenBCI system required careful preparation, including proper electrode placement, stable electrode–scalp contact, reference electrode positioning, and minimal participant movement to ensure reliable signal recording.
- Motion artifacts, ocular movements, muscular activity, poor electrode contact, and environmental noise affected EEG signal quality, requiring effective preprocessing.
- Extracting multiscale entropy features across multiple scale factors increased the computational load and required suitable testing procedures.
- The high-dimensional feature space increased the risk of overfitting, necessitating feature selection and optimization for better classification performance.

To address these challenges, suitable methodologies were developed and are discussed in the forthcoming sections.

## 3 Materials and methods

The current research analyses the cognitive activity between sports and non-sports individuals using EEG and selects the features that have a high impact on cognitive performance. The analysis is aimed at ranking and selecting the major contributing EEG feature subset to enable an effective comparison and categorization at the group level.

### 3.1 Subjects and data acquisition

The dataset for the present study was collected from Hindustan Institute of Technology and Science (HITS), Chennai, Tamil Nadu, India. Institutional ethical approval was obtained. The approval number is HITS/SPESS/I/22. The subjects comprised 50 healthy individuals, each from sports and non-sports group. The sports group was restricted to healthy individuals who were under active training, had a competitive experience in the State, National or All India Inter University level and were not injured in the near past. The non-sports group consisted of healthy people who did not undergo any physical exercise regularly. The sports group consisted of 20, 10, 10, 5, and 5 men soccer players, men basketball players, women basketball players, men volleyball players, and men table tennis players, respectively. The age of men and women subjects was 19 to 23 years; both men and women had a height of 168 to 185 cm and 152 to 170 cm, respectively. In both groups, the process of selection was based on availability, willingness and basic health checkup. Informed consent was obtained prior to data collection through writing. Figure 1 shows the entire methodology of the planned study. EEG signals were recorded using small, circular, reusable dry-pin electrodes mounted on an OpenBCI Ultracortex Mark IV headset. These electrodes established direct scalp contact without conductive gel. The recording electrodes were positioned at F3–A1, F4–A2, P3–A1, and P4–A2 as per International 10–20 EEG electrode system, with A1 and A2 serving as reference locations. The electrode signals were acquired using a high-input-impedance OpenBCI board and digitized at a sampling frequency of 256 Hz. All participants performed a mental arithmetic task in a relaxed state with eyes open and no movements. Before each recording, electrode contact quality and signal stability were checked to reduce contact-related noise, movement artifacts, and power-line interference. The acquired EEG signals were reviewed and validated for their quality and suitability for cognitive analysis by a medical expert.

**Figure 1:**
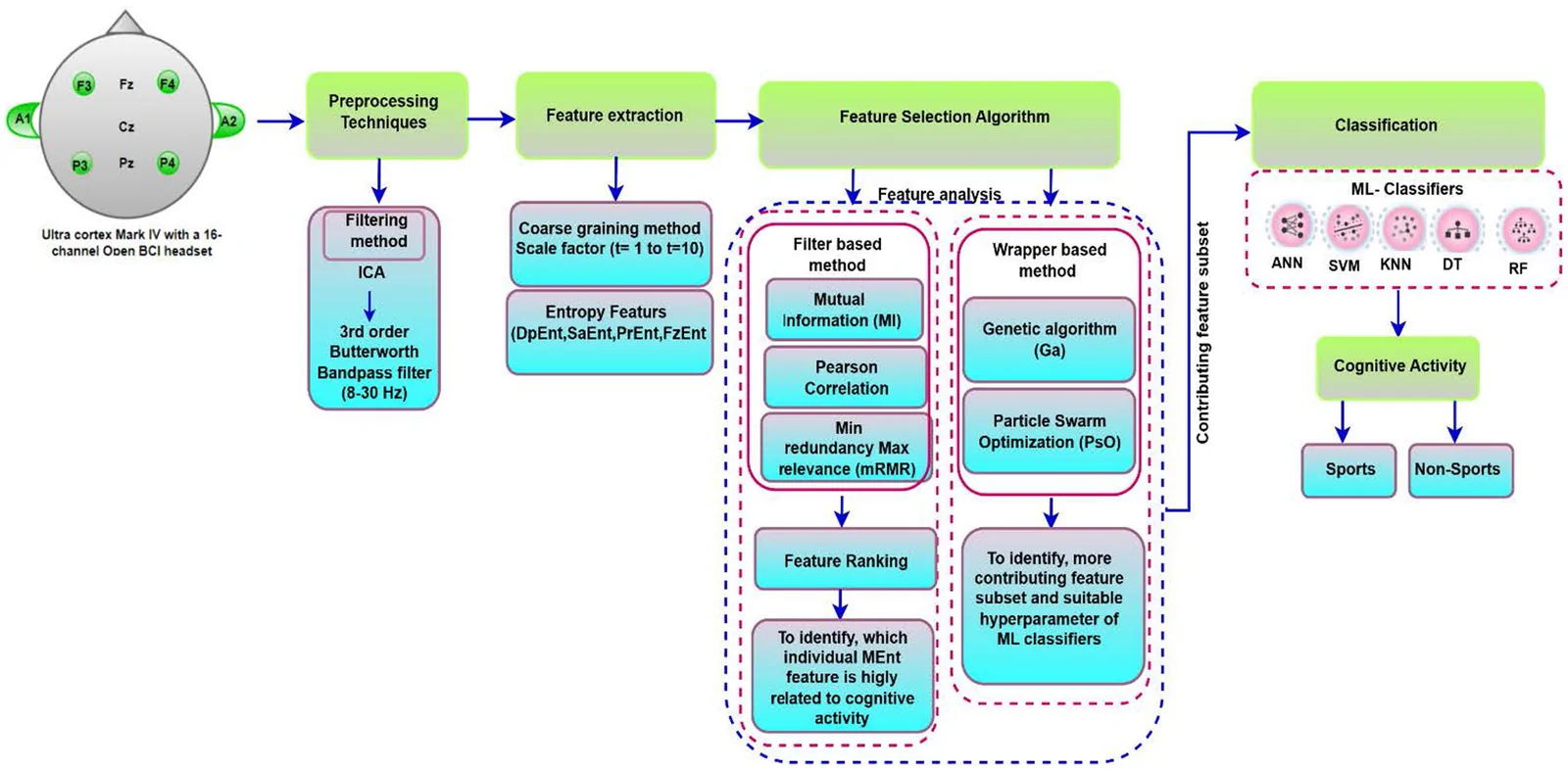
Flow diagram of cognitive activity analysis. The pipeline includes EEG preprocessing, multiscale entropy feature extraction, filter- and wrapper-based feature selection, and ML-based classification for differentiating the cognitive activity between sports and non-sports individuals.

### 3.2 Preprocessing

Raw EEG signals often have various noise factors and artifacts in them that make it hard to see the real neural activity. Removing these unwanted components improves signal quality, enhances interpretability and ensures that the data reflects brain activity. As evident from the studies, the filtering methods were used for the EEG preprocessing in the cognitive activity analysis (Keerthika and Kiruthika, 2024; Onate et al., 2000; Dai et al., 2019; Keerthika and Kiruthika, 2026a). These preprocessing techniques effectively suppress non-neural interference while preserving relevant EEG information, thereby enhancing signal quality for reliable cognitive analysis. In the present study, filtering method which consists ICA was used to remove the eyeblink artifacts, followed by third order butterworth bandpass filter to retain the specific frequency range (8–30 Hz) of alpha and beta bands for analyzing the cognitive functions. These methods effectively minimized the artifact, noise and unwanted components.

Signal to Noise Ratio (SNR) and Peak Signal to Noise Ratio (PSNR) (Jung et al., 2000) were computed using the complete 1-min EEG recordings acquired at 256 Hz from four electrodes (*M* = 15, 360 samples per channel) using Equations 1, 2. An independently denoised EEG signal *s*_*j*_[*k*] was treated as the clean reference, and controlled 50-Hz interference was added to generate the noisy test signal. After applying the preprocessing filtering method, the recovered signal *y*_*j*_[*k*] was compared with the clean reference, and the residual error was defined in the Equation 3.

$$\begin{array}{l} \begin{array}{c} SNR_{j}\;=\;10\log_{10}\left(\frac{\sum_{k\;=\;1}^{M}s_{j}^{2}\left[k\right]}{\sum_{k\;=\;1}^{M}\varepsilon_{j}^{2}\left[k\right]}\right)\;dB, \end{array} \end{array}$$ (1)

$$\begin{array}{l} \begin{array}{c} PSNR_{j}\;=\;10\log_{10}\left(\frac{A_{j}^{2}}{MSE_{j}}\right)\;dB,\;MSE_{j}\;=\;\frac{1}{M}\sum_{k\;=\;1}^{M}\varepsilon_{j}^{2}\left[k\right], \end{array} \end{array}$$ (2)

$$\begin{array}{l} \varepsilon_{j}\left[k\right]\;=\;s_{j}\left[k\right]-y_{j}\left[k\right] \end{array}$$ (3)

*wherej*−*Index representing the signal of EEG channelk*−*discreatetime sample IndexMSE*_*j*_−*Mean squared reconstruction error*$\varepsilon_{j}\left[k\right]-Error\;of\;j^{th}\;signal/component\;at\;the\;k^{th}sample\;$*s*_*j*_[*k*]−*Original, reference, of desired value of the* *j*^*th*^ *signal at sample* *ky*_*j*_[*k*]−*Measured, reconstructed, filtered, of estimated value of the* *j*^*th*^*signal at sample* *k*

### 3.3 Feature extraction

Feature extraction helps identify the relevant information contained in EEG signals. Entropy is a quantitative measure of complexity of the EEG signal which describes the irregularity and unpredictability of neural activity (Zavala-Yoe and Ramirez-Mendoza, 2017). More diverse and flexible neural dynamics, associated with higher entropy, may reflect greater cognitive engagement and adaptive information processing. Conversely, lower entropy means more regularity in the brain and may be linked to less cognitive processing or fatigue (Chueh et al., 2025; Nawaz et al., 2022). Nevertheless, the direction of entropy change is task-dependent, as EEG complexity may either increase or decrease according to the nature and intensity of the cognitive demand (Keshmiri, 2020). For instance, increased EEG complexity has been reported under greater working-memory load (Natarajan et al., 2004). Therefore, entropy-based features were analyzed in the multiscale factors by using a method called coarse graining method. When calculated across coarse-grained EEG signals, entropy therefore captures neural dynamics at multiple temporal scales and can be interpreted as an indicator of the brain's capacity for dynamic information processing and integration. Various studies analyzed the cognitive function in sports using MEnt (Eroglu et al., 2020; Lu et al., 2024; Sukriti and Mitra, 2021b). It serves as a useful indicator to analyze the cognitive activity in precision tasks. The coarse-graining method transforms the pre-processed EEG signal into several components at different temporal scales (Ramyarangsi et al., 2024; Costa et al., 2002). It captures changes in EEG signal complexity across different time scales, which can support the classification of cognitive states in sports and non-sports individuals. For each scale factor, the original EEG signal is divided into non-overlapping segments, and the samples within each segment are averaged to construct a coarse-grained time series. Each preprocessed EEG signal was coarse-grained using scale factors *t* = 1*t* = 1 to 15 by averaging consecutive non-overlapping samples. The values increased gradually with the increase of the scale factor, reached a maximum at *t* = 10, and then dropped slightly. Thus, *t* = 1 to *t* = 10 scales were selected (Costa et al., 2005). MSaEnt, MFzEnt, MPrEnt, and MDpEnt were then calculated separately from the signal obtained at each scale. Algorithm-specific checks were applied at every scale. For MSaEnt, nonzero template matches at *m* and *m*+1 were confirmed with *Y*^*m*^>0 and *Y*^*m*+1^>0. For MFzEnt, positive and finite mean fuzzy similarities were verified. For MPrEnt, sufficient embedding vectors and valid ordinal-pattern were checked using *N*_*t*_−(*m*−1)*d* and pattern frequencies. For MDpEnt, the pattern-space size, dispersion-class mapping, and class indices within $c^{m}<N_{t}\;$and class indices 1 to *c* were verified. Finally, all entropy outputs were checked for NaN and infinite values before further analysis (Keerthika and Kiruthika, 2026b; Delgado-Bonal and Marshak, 2019; Azami et al., 2017a,b).

#### 3.3.1 Sample entropy (SaEnt)

ApEnt is an early complexity measure which measures regularity in time series by checking whether similar patterns of length m remain similar when extended to *m* + 1 (including self-matches) (Pincus et al., 1991). SaEnt was later proposed as an extension of ApEnt by removing self-matches, which reduces the bias of the estimation and provides more consistent estimates, especially for the physiological signals of finite length (Richman and Moorman, 2000; Pelc et al., 2022). For a time, series X = {x1, x_2_, ..., xL}, embedding vectors of length m are constructed in the Equation 4.

$$\begin{array}{l} X_{i^{m}}=\;\left[x_{i},\;x_{i+1},\;...,\;x_{i+m^{-1}}\right],\;1\;\leq \;i\;\leq \;L\;-\;m\;+\;1 \end{array}$$ (4)

$$\begin{array}{l} SaEnt\left(X,m,r\right)\;=\;-\frac{\ln\;Y^{m+1}}{Y^{m}} \end{array}$$ (5)

Two vectors are considered matched when $d_{ij}^{m}$ ≤ r, where r is the tolerance threshold. If *Y*^*m*^ and *Y*^*m*+1^ denote the average probabilities of matching distinct vector pairs of lengths m and *m* + 1, respectively, SaEnt is calculated using Equation 5. A lower SaEnt value indicates more repetitive and predictable temporal patterns, whereas a higher value indicates greater irregularity and complexity. For multiscale analysis (Song and Liò, 2010), the original time series is coarse-grained at each scale factor t.

$$\begin{array}{l} G_{s}^{t}\;=\;\frac{1}{t}\sum_{i\;=\;\left(s-1\right)t}^{st}x_{i}\;,\;1\leq s\leq |\frac{L}{t}| \end{array}$$ (6)

*where*$G_{s}^{t}$*-coarse grained time seriest-scale factors-index of the coarse-grained data pointx*_*i*_*-original time series dataL-length of the original series data*At *t* = 1, the coarse-grained series is identical to the original signal. At *t* > 1, consecutive non-overlapping groups of t samples are averaged. Combining Equations 5, 6 yields the formula for MSaEnt, as depicted in Equation 7.

$$\begin{array}{l} MSaEnt\left(X,t,m,r\right)\;=\;SaEnt\left(G_{s}^{t},m,r\right) \end{array}$$ (7)

*wherem*−*Embedding dimensionr*−*tolerence*

Previous studies have reported statistically reliable SaEnt estimation using *m* = 1 or *m* = 2, with *r* commonly ranging from 0.10 to 0.25 times the standard deviation (SD) of the original time series (Pelc et al., 2022; Song and Liò, 2010; Ahmed and Mandic, 2012). Based on these recommendations, a parameter-sensitivity analysis was conducted using *m* = 2 and *m* = 3 in combination with *r* = α × SD(*X*), where α = 0.10, 0.15, 0.20, and 0.25. As shown in Figure 2, SaEnt decreased systematically with increasing *r*, while comparable trends were maintained for both embedding dimensions. Although *m* = 1 can provide statistically valid estimates, it compares individual samples and provides limited representation of temporal organization. Conversely, *m* = 3 represents longer patterns but reduces the number of available matches, particularly after multiscale coarse-graining. Therefore, *m* = 2 was retained to represent short temporal patterns while preserving sufficient matching vectors. The sensitivity to small temporal variations was balanced against the stability of the estimates by selecting the intermediate tolerance *r* = 0.20 × SD(*X*).

**Figure 2:**
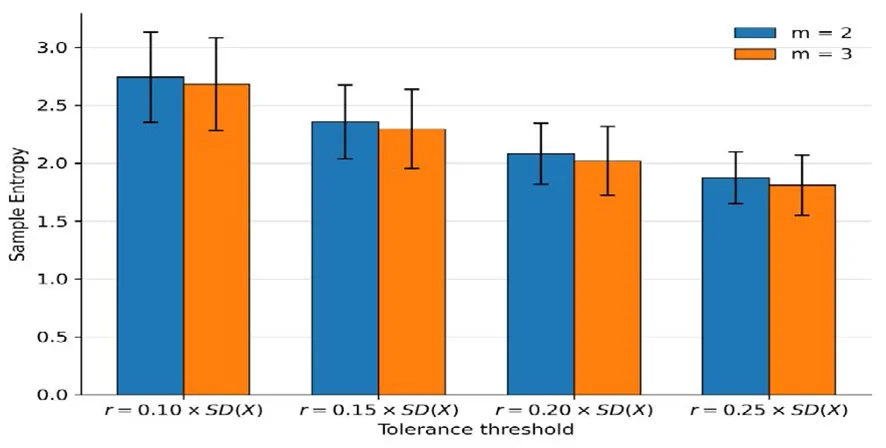
Mean ± SD of SaEnt. SaEnt obtained using *m* = 2 and *m* = 3, with *r* = α × SD(*X*) and α = 0.10,0.15,0.20, and 0.25. Bars represent the participant mean ± SD. SaEnt decreased progressively with increasing *r*, while similar trends were maintained for both embedding dimensions.

Further, to substantiate the importance of the selection of the combination of parameters, the SaEnt features were fed into the SVM model and accuracy was computed. Table 1 complements this signal-level analysis by showing the corresponding SVM classification performance. Among all parameter combinations, *m* = 2 and *r* = 0.20 × *SD*(*X*)achieved the highest accuracy of 93.33%. Therefore, this combination was retained because it provided stable SaEnt estimation and the best classification performance.

**Table 1:** Comparative analysis of SaEnt parameter selection.

SaEnt–parameter analysis				
Embedding dimension (*m*)	Accuracy %			
	Tolerance			
	*r* = 0.10	*r* = 0.15	*r* = 0.20	*r* = 0.25
1	83.33	83.33	80	76.67
2	86.67	86.67	93.33	90
3	90	86.67	91.61	86.67

Accuracy was evaluated using SVM for each *m*-*r* combination; *m* = 2 and *r* = 0.20 × *SD*(*X*) achieved the highest accuracy of 93.33%.

#### 3.3.2 Fuzzy entropy (FzEnt)

FzEnt was developed to improve the stability of entropy estimation by replacing the binary matching criterion used in SaEnt with a continuous fuzzy membership function (Xiang et al., 2015). It measures time-series regularity by assigning a gradual degree of similarity to each pair of embedding vectors. Consequently, small variations near the tolerance boundary do not cause an abrupt transition between matched and unmatched patterns. For the embedding vectors defined in Equation 8, the local mean of each vector is removed before the similarity calculation. The maximum absolute component-wise difference between two distinct mean-adjusted vectors is denoted by $d_{ij^{m}}$. Their similarity is calculated using the exponential fuzzy membership function in Equation 8.

$$\begin{array}{l} D\left(n,r\right)\;=\;\exp\left(-\frac{\left(d\right)n}{r}\right),\;\;i\;\neq \;j \end{array}$$ (8)

$$\begin{array}{l} FzEnt\left(X,m,r,N\right)\;=\;-\frac{\ln\left(\Phi^{m+1}\left(n,r\right)\right)}{\Phi^{m}\left(n,r\right)} \end{array}$$ (9)

If Φ^*m*^(*n*,*r*) and Φ^*m*+1^ *n*,*r*) denote the average fuzzy similarities obtained for embedding dimensions m and *m* + 1, respectively, FzEnt is calculated using Equation 9. *whereX-time series datam-embedding dimensionr-tolerance parametern-exponent controlling the gradient of the fuzzy membership functionN-total number of samples in the time series data *${\mathrm{XD}}_{ij^{m}}$*(n,r)-fuzzy similarity between the vectors i and *${\mathrm{jd}}_{ij^{m}}$*-distance between the vectors i and j*

For multiscale analysis, the coarse-grained series G^*t*^ obtained using Equation 6 is used to calculate FzEnt separately at each scale factor. Multiscale Fuzzy Entropy (MFzEnt) is expressed using Equation 10.

$$\begin{array}{l} MFzEnt\left(X,t,m,r\right)\;=\;FzEnt\left(G_{s}^{t},m,r\right) \end{array}$$ (10)

*t* = 1, MFzEnt is equivalent to the FzEnt of the original preprocessed signal. At *t* > 1, it represents the fuzzy pattern similarity of the progressively coarse-grained signal. The calculation of MFzEnt is mainly influenced by the embedding dimension m, similarity tolerance *r*, fuzzy power *n*, and scale factor *t*. The embedding dimension determines the length of the patterns reconstructed from the EEG signal. The similarity tolerance r and fuzzy power *n* determine the width and gradient of the fuzzy membership function. Based on previous recommendations, *m* is commonly set to 2, while n is varied between 1 and 3 and *r* is generally selected between 0.10 and 0.25 times the standard deviation of the original signal (Azami et al., 2017a; Xiang et al., 2015).

As illustrated in Figure 3a, value of m is fixed as 2, *r* is fixed as 0.20 and the impact of varying n is studied. It is inferred that *n* = 1 produces a rapid reduction in fuzzy similarity and may increase sensitivity to small distance variations, whereas *n* = 3 produces a broader transition and may reduce sensitivity to subtle signal differences. Therefore, *n* = 2 was selected to provide an intermediate and gradual similarity transition. Similarly, the impact of varying *r* is studied by fixing the values of *m* and *n* as 2 as shown in Figure 3b. A small *r* produces a narrow similarity boundary and may make entropy estimation sensitive to noise, whereas a large r produces a broad boundary that may suppress relevant signal variations. The selected value *r* = 0.20 × SD provides a balance between noise sensitivity at smaller *r* values and loss of detailed signal information at larger values. In the present study, MFzEnt was calculated using an embedding dimension of *m* = 2, fuzzy power *n* = 2, and similarity tolerance *r* = 0.20 × SD of each EEG segment.

**Figure 3:**
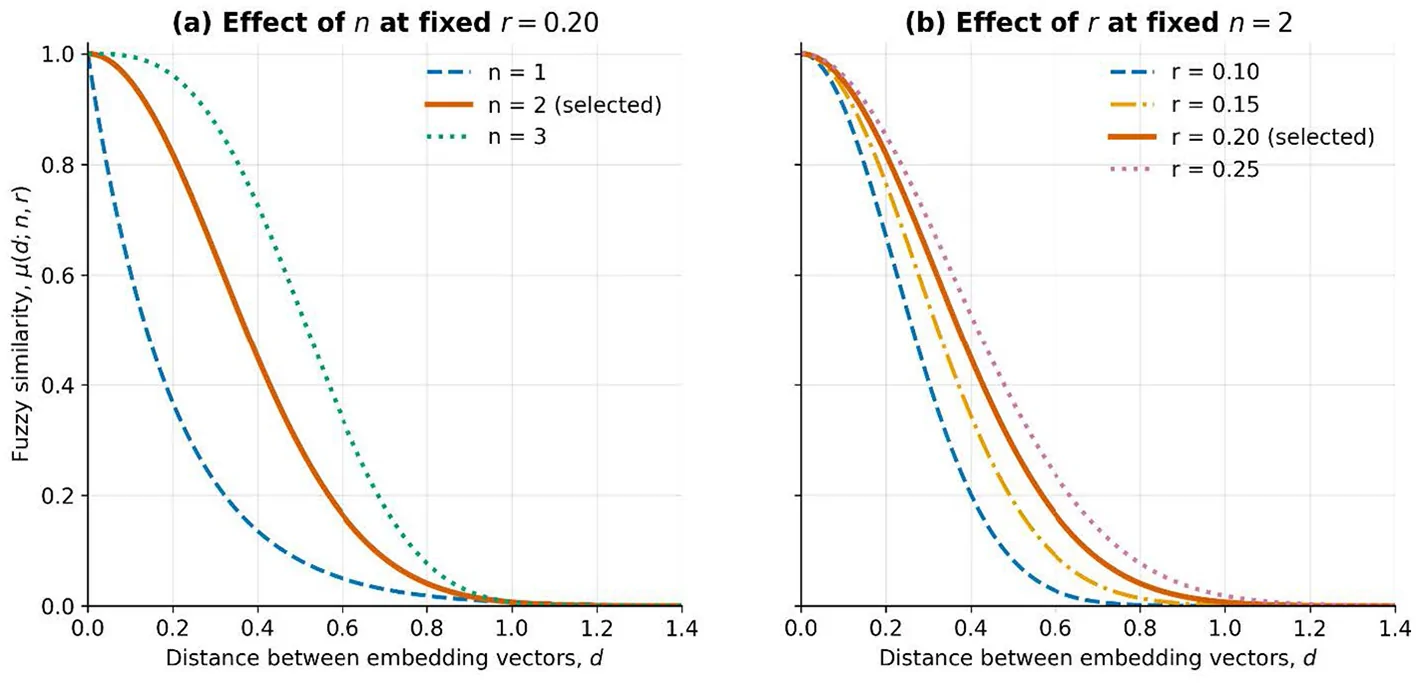
Combined influence of fuzzy exponent and tolerance on the exponential fuzzy membership function. **(a)** effect of fuzzy power n at fixed *r* = 0.20, with *n* = 2 selected, **(b)** effect of similarity tolerance r at fixed *n* = 2, with *r* = 0.20 × SD selected.

#### 3.3.3 Permutation entropy (PrEnt)

PrEnt measures the temporal complexity through the analysis of the relative ordering of the samples in the embedded vectors. Each vector is mapped to an ordinal pattern, and the occurrence probabilities of all observed patterns are used to calculate entropy. Multiscale Permutation Entropy (MPrEnt) applies this procedure to coarse-grained signals at successive scale factors, thereby characterizing changes in ordinal dynamics across multiple temporal resolutions (Bandt and Pompe, 2002). PrEnt is defined in Equation 11.

$$\begin{array}{l} PrEnt\left(X,m,d\right)\;=\;-\;\sum_{\lambda_{i\;=\;1}\;}^{m}p\left(\lambda_{i}\right)\ln\left(p\left(\lambda_{i}\;\right)\right)\; \end{array}$$ (11)

*whereX* = *data lengthm* = *Embedding dimensiond* = *delayp*(λ_*i*)_ = *Probability of different permutations of the sequence of length m derived from the time seriesX. Each* λ_*i*_ *corresponds to a unique arrangement of the data points*

It quantifies how frequently each permutation pattern appears. MPrEnt is calculated by combining Equations 6, 11, resulting in Equation 12.

$$\begin{array}{l} MPrEnt\left(X,t,m,d\right)\;=\;PrEnt\left(G_{s}^{t},m,d\right) \end{array}$$ (12)

The reliability of PrEnt depends primarily on the embedding dimension (*m*), time delay (*d*), and available signal length. Previous studies have commonly investigated embedding dimensions within *m* = 3 to 7, with a unit delay *d* = 1 when the temporal ordering of consecutive samples is of interest (Bandt and Pompe, 2002; Franka et al., 2024). In the present study, parameter sensitivity was evaluated using *m* = 2-5 at *d* = 1 and *d* = 1-3 at *m* = 3 across scale factors *t* = 1 to 10. As shown in Figure 4a, larger *m* values produced lower normalized PrEnt at smaller scales because the number of possible ordinal patterns increases factorially as *m*!. Although *m* = 2 produced maximum normalized PrEnt, it represents only 2! = 2 patterns and provides limited temporal information. Therefore, *m* = 3, representing 3! = 6 ordinal patterns, was selected. Figure 4b shows higher PrEnt for *d* = 2 and *d* = 3; however, *d* = 1 was retained to preserve consecutive-sample relationships. For a coarse-grained signal of length *N*_*t*_ = ⌊*L*/*t*⌋, the number of available embedding vectors is *N*_*vec*_ = *N*_*t*_−(*m*−1)*d*. Accordingly, *m* = 3 and *d* = 1 were retained because they provided a practical balance between temporal-pattern representation, complete ordinal-pattern coverage, and reliable PrEnt estimation across the investigated scales.

**Figure 4:**
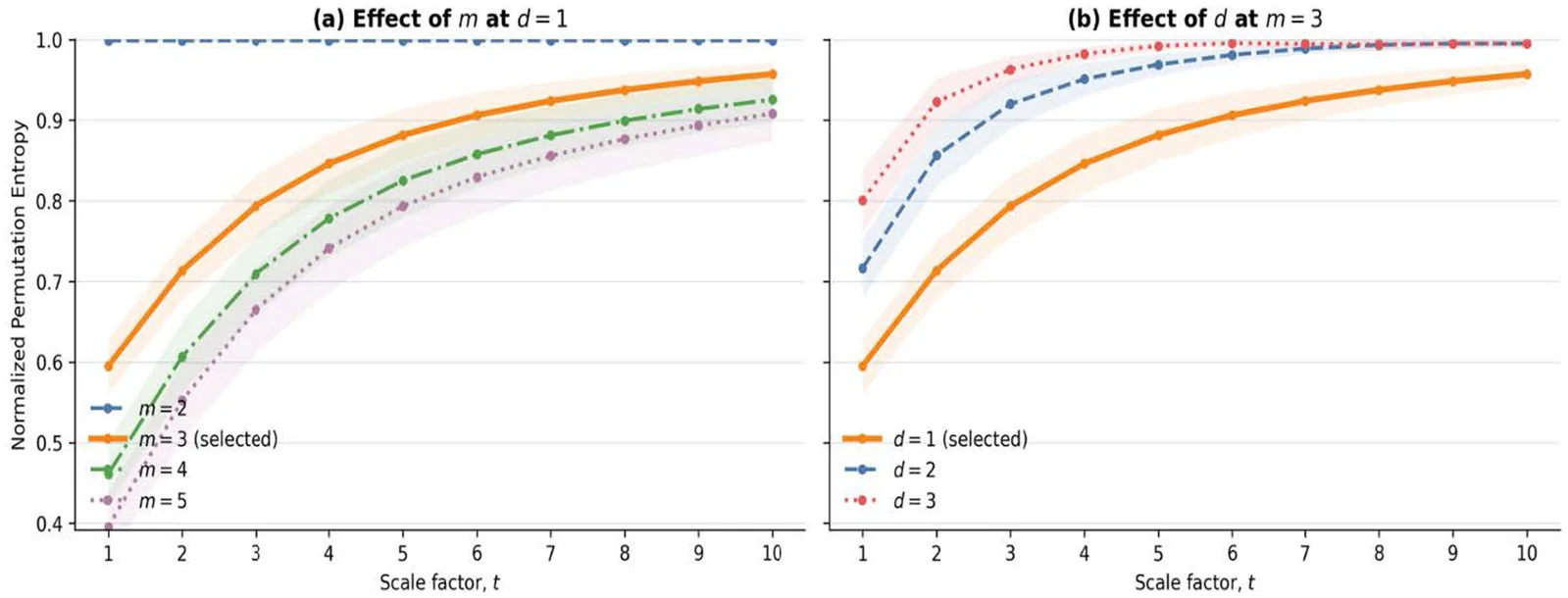
**(a, b)** Sensitivity of normalized PrEnt to embedding dimension and time delay across scale factors *t* = 1 to 10. Curves and shaded regions represent participant mean ± SD. The selected parameters and are highlighted.

#### 3.3.4 Dispersion entropy (DpEnt)

DpEnt was developed to address the ambiguity caused by equal-valued samples in the ordinal-pattern representation used by PrEnt. It maps signal amplitudes into discrete classes and evaluates the probability distribution of the resulting dispersion patterns. Consequently, DpEnt retains amplitude-related information and provides a stable measure of time-series complexity at a single temporal scale (Rostaghi and Azami, 2016). DpEnt is defined in Equation 13.

$$\begin{array}{l} DpEnt\left(X,m,d,c\right)\;=\;-\underset{r}{\sum }y\left(r\right).\ln\left(y\left(r\right)\right) \end{array}$$ (13)

*whereX-data lengthm-embedding dimensiond-delayc-Classy(r)-*p*robability of occurrence of the r-th class*

The MDpEnt is derived by combining Equations 6, 13, resulting in the expression given in the Equation 14.

$$\begin{array}{l} MDpEnt\left(X,m,c,d,t\right)\;=\;DpEnt\left(G_{s}^{t},m,c,d\right) \end{array}$$ (14)

Figure 5a shows that the MDpEnt trajectories changed with increasing *m*. This is due to the increase in the number of possible dispersion patterns from 3^2^ = 9 at *m* = 2 to 3^4^ = 81, at *m* = 4. Figure 5b demonstrates that increasing *c* produced progressively higher entropy values, while *c* = 3 retained an intermediate amplitude resolution without unnecessarily expanding the pattern space. Figure 5c shows that larger delays caused the entropy estimates to approach the normalized upper limit more rapidly, whereas *d* = 1 preserved a clearer scale-dependent trajectory. Therefore, *m* = 2, *c* = 3, *d* = 1 provided a practical balance between amplitude resolution, temporal representation, pattern-space size, and scale sensitivity. For reliable probability estimation, the number of possible dispersion patterns should satisfy $c^{m}<[L/t_{\max}$] (Sukriti and Mitra, 2021b; Rostaghi and Azami, 2016; Costa et al., 2002), where L is the original signal length and [*L*/*t*_max_] is the length of the shortest coarse-grained series. In the present study, each 60-s EEG segment contained *L* = 15,360 samples. At *t*_max_ = 10, the shortest coarse-grained series contained [15,360/10] = 1,536 samples. Parameter combinations *m* = 2–4 and *c* = 2–6 was examined, producing between 2^2^ = 4 and 6^4^ = 1,296 possible dispersion patterns. Therefore, all tested combinations satisfied the above requirement. Based on the sensitivity analysis shown in Figures 5a–c, *m* = 2, *c* = 3, and *d* = 1 was selected. This combination produces nine possible dispersion patterns, provides adequate amplitude resolution, preserves consecutive-sample relationships, and retains sufficient observations for reliable probability estimation across scales *t* = 1 to10.

**Figure 5:**
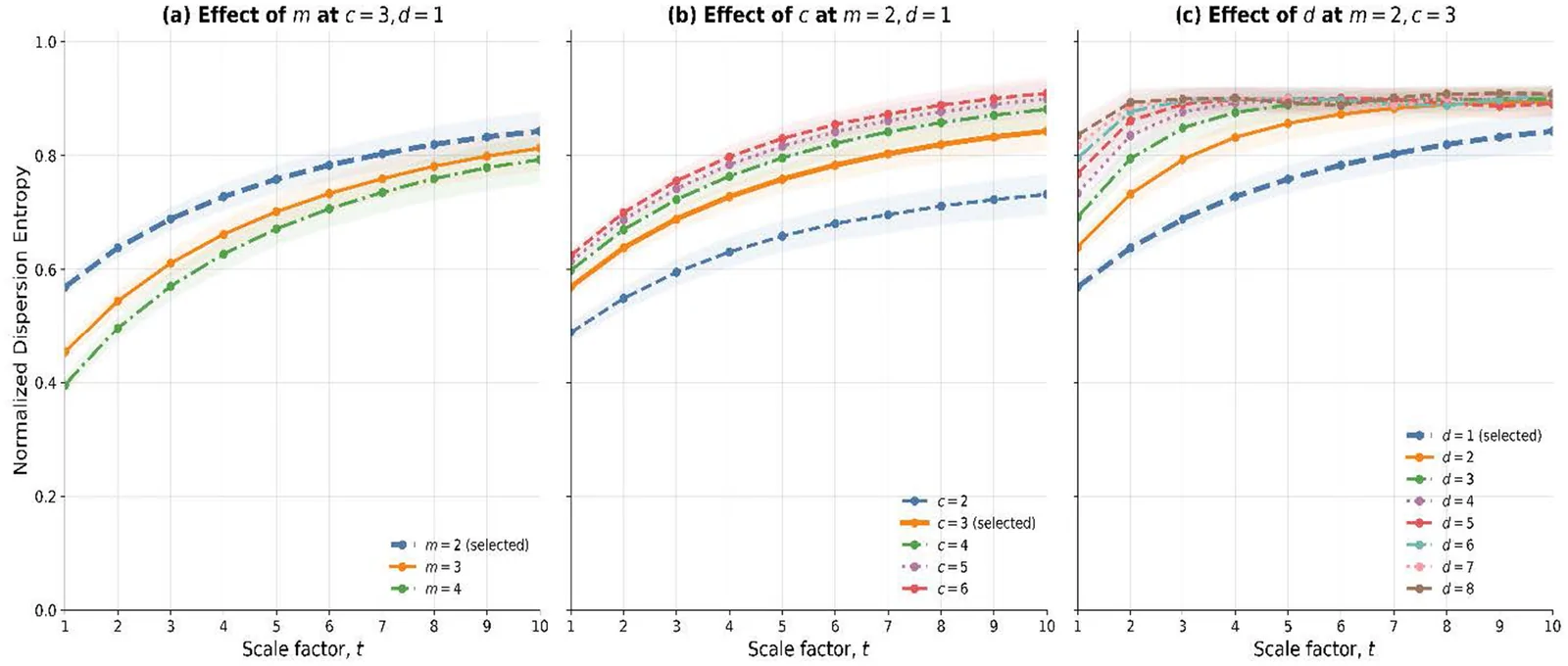
Sensitivity of normalized Multiscale DpEnt to **(a)** embedding dimension *m*, **(b)** number of amplitude classes *c*, and **(c)** time delay *d*, across scale factors *t* = 1 to 10. Curves and shaded regions represent participant mean ± SD. The selected parameters *m* = 2, *c* = 3, and *d* = 1 is highlighted.

This produced 40 features per electrode and a total of 160 MEnt features from F3, F4, P3, and P4 as depicted in Figure 6. However, when multiple MEnt features are considered at once, it is not clear which of them contributes most to describing cognitive activity, and the increased dimensionality, raises computational load and the risk of overfitting. To address this, the complete feature set was not used directly instead, feature selection algorithms were applied to identify a smaller group of informative and non-redundant features for further analysis and classification.

**Figure 6:**
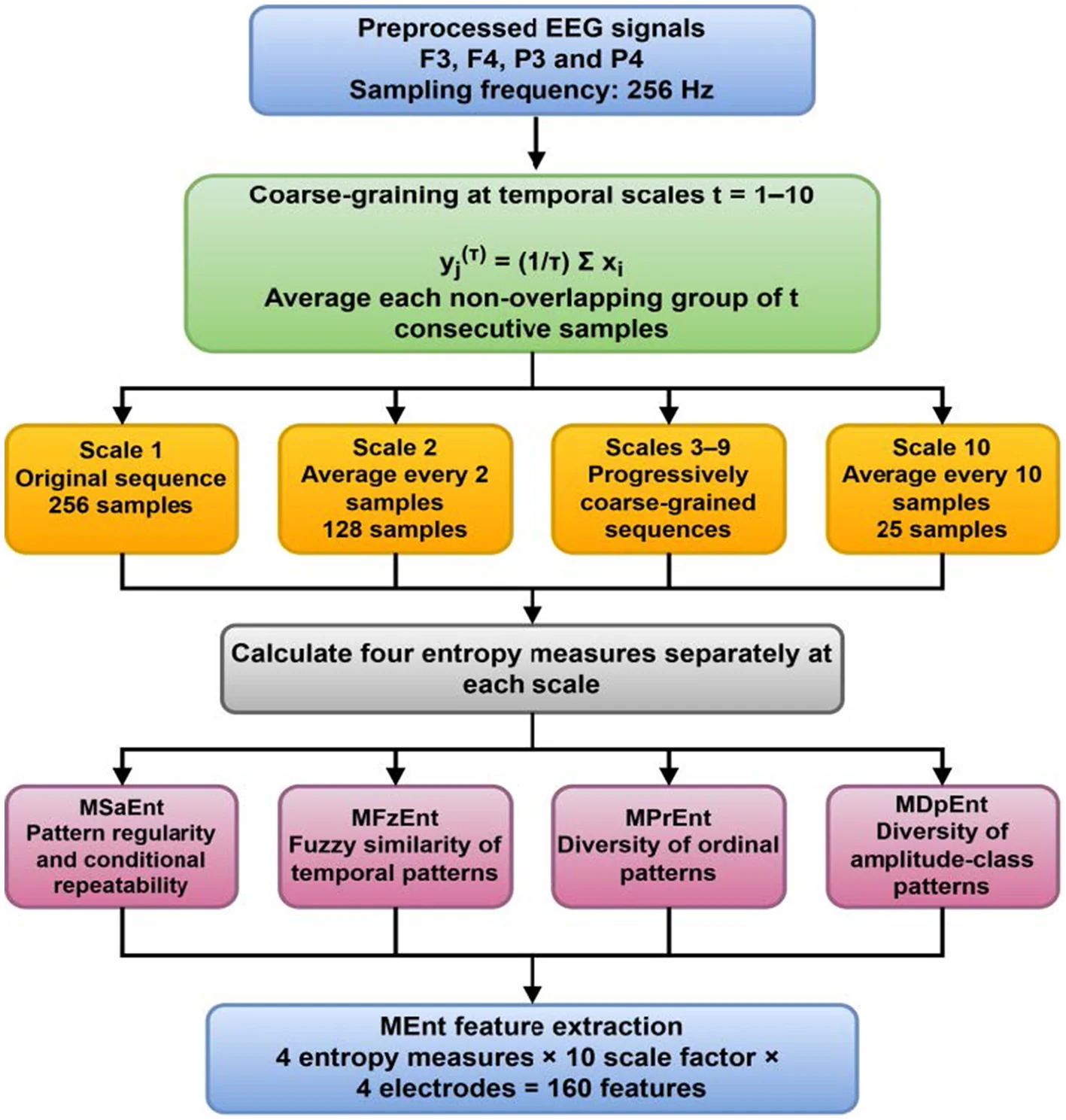
MEnt feature extraction using coarse graining method. This process resulted in 40 features for each electrode, culminating in a total of 160 MEnt features derived from the electrodes F3, F4, P3, and P4.

### 3.4 Feature selection

Feature selection is used as a separate step to organize and refine the extracted EEG features before classification. Instead of using all the extracted features, it helps to identify the more contributing features for the further analysis. Several feature selection methods were used, such as filter and wrapper based. The filter-based method prioritizes features based on statistical criteria. This method is to identify significant features that contribute unique information (Xu et al., 2023; Chen et al., 2023). Wrapper-based methods integrate optimization techniques to evaluate feature subsets based on their direct impact on classifier performance. Wrapper-based methods, including Ga, Sequential Forward Selection (SfS), and PsO have been widely utilized for EEG feature analysis (Ghayab et al., 2016; Yedukondalu et al., 2025; Ihianle et al., 2024). In the first stage, filter-based methods such as mRMR, Pearson correlation and MI were used to rank and screen individual features. In the second stage, wrapper methods such as Ga, and PsO were used to explore alternative subsets in the feature space. These combined procedures help to remove redundant features, lower computational load and highlight the EEG features that contribute more for the cognitive activity analysis.

#### 3.4.1 Filter based methods

Filter-based techniques consider each feature independently, and they are only based on the statistical relationship between that feature and the class label. This method provides a simple way of filtering large sets of features and eliminates variables, which show only weak correlations with cognitive activity. In this study, the relationship between EEG features and the class label, which can be either sports or non-sports was analyzed.

##### 3.4.1.1 Pearson correlation

The Pearson correlation (Pawan, 2023) is used to measure the magnitude of the linear relationship between each EEG feature and the label of the class. Equation 15 gives the average value of the feature x and the average value of the class label y of all N samples. In Equation 16, the sample covariance between the feature and the class label is calculated.

$$\begin{array}{l} \begin{array}{c} \overset{-}{a}\;=\;\frac{1}{N}\sum_{k\;=\;1}^{N}a_{k}\;and\;\overset{-}{b}\;=\;\frac{1}{N}\sum_{k\;=\;1}^{N}b_{k}. \end{array} \end{array}$$ (15)

$$\begin{array}{l} \begin{array}{c} Cov\left(a,b\right)\;=\;\frac{1}{N-1}\sum_{k\;=\;1}^{N}\left(a_{k}-\overset{-}{a}\right)\left(b_{k}-\overset{-}{b}\right). \end{array} \end{array}$$ (16)

*wherea*_*k*_ = *value of the considered EEG feature for sample k*.*b*_*k*_ = *class label for sample k*(1 = *sports*, 0 = *non*−*sports*)*N* = *total number of samples*$\overset{-}{a}\;=\;Mean\;of\;the\;feature\;values\;$$\overset{-}{b}\;=\;Mean\;of\;the\;class\;labels\;$*Cov*(*a, b*) = *Sample covariance between feature and label* Equation 16 calculates Cov(*a*,*b*), which shows whether greater feature values are more likely to co-occur with sports (*b*_*k*_ = 1) or non-sports (*b*_*k*_ = 0).

The standard deviation of the feature and that of the class label are

$$\begin{array}{l} \begin{array}{c} \sigma_{a}\;=\;\sqrt{\frac{1}{N-1}\sum_{k\;=\;1}^{N}{\left(x_{a}-\overset{-}{a}\right)}^{2}},\;and\;\sigma_{b}\;\\ =\;\sqrt{\frac{1}{N-1}\sum_{k\;=\;1}^{N}{\left(b_{k}-\overset{-}{b}\right)}^{2}}. \end{array} \end{array}$$ (17)

*where* σ_*a*_, σ_*b*_ = *SD of feature and label*.

Equation 17 produces the spread of the feature values around their mean, σ_a_ and the spread of the class labels around their mean, σ_b_. The correlation coefficient r_ab_ in Equation 18 is the covariance divided by the product of the two standard deviations such that r_ab_ is always between −1 and +1.

$$\begin{array}{l} \begin{array}{c} r_{ab}\;=\;\frac{Cov\left(a,b\right)}{\sigma_{a}\sigma_{b}}. \end{array} \end{array}$$ (18)

*wherer*_*ab*_ = *Pearson correlation*σ_*a*_, σ_*b*_ = *SD of feature and label*

Values close to zero are the ones which have a negligible and non-linear relationship with the class label. The absolute correlation, r of the features (*r*_*ab*_) is used in the univariate selection step where high valued features are kept as more important in differentiating the cognitive activity between the groups.

##### 3.4.1.2 Mutual information (MI)

MI (Arslan, 2018) is used to remove irrelevant or redundant features in the original set of features. It measures the relevance of two random variables. In the feature selection phase, A is the EEG feature vector and B is the class label. When there is independence between a feature and class label, MI is zero; larger MI values indicate the presence of a stronger dependency and as such, make a feature more informative in the subset. I(A:B) is an expression of the decrease in the total uncertainty when the class is viewed together with the feature, as compared with treating them individually. Basic characteristics of MI such as non-negativity and symmetry are presented in Equations 19, 20.

$$\begin{array}{l} I\left(A:B\right)\geq 0, \end{array}$$ (19)

$$\begin{array}{l} I\left(A:B\right)\;=\;I\left(B:A\right) \end{array}$$ (20)

The feature selection using MI is conducted on each of the EEG features (A) against the class label (B) and the features are ranked based on MI values. The ones with higher I(A:B) are captured to further analysis and those with very low MI are deemed irrelevant and therefore are ignored.

##### 3.4.1.3 Minimum redundancy maximum relevance (mRMR)

mRMR (Peng et al., 2005; Al-Nafjan, 2022) is a feature selection method used to identify EEG features that are highly informative for classification with minimum repeated information. In this method, the original EEG feature set is evaluated based on two important criteria such as relevance and redundancy. Relevance, measures how strongly each EEG feature is associated with the class label, whereas redundancy measures how much similar or repeated information exists between the candidate feature and the already selected features. In the feature selection process, let *X* represent the EEG feature and *Y* represent the class label. The relevance between the EEG feature and the class label is measured using MI method, as shown in the Equation 21, where *I*(*X*:*Y*) indicates how much information the feature *X* provides about the class label *Y*.

$$\begin{array}{l} \begin{array}{c} Relevance\;=\;I\left(X:Y\right)\;\;\;\;\;\;\;\; \end{array} \end{array}$$ (21)

$$\begin{array}{l} \begin{array}{c} \;\;Redundancy\;=\;I\left(X_{i}:X_{j}\right) \end{array} \end{array}$$ (22)

After calculating relevance, redundancy is estimated between the EEG feature *X*_*i*_ and the already selected EEG feature *X*_*j*_, as depicted in the Equation 22. *I*(*X*_*i*_:*X*_*j*_) measures the amount of overlapping information between two features. If two features carry similar information, the redundancy value becomes high. Such features may not add much new information to the classification process. Finally, the mRMR score is calculated by subtracting redundancy from relevance as shown in the Equation 23.

$$\begin{array}{l} \begin{array}{c} mRMRScore\;=\;I\left(X:Y\right)-I\left(X_{i}:X_{j}\right) \end{array} \end{array}$$ (23)

Features with higher mRMR scores are considered more useful because they have strong relevance with the class label and low redundancy with other selected features. The top-ranked features were selected as they provide better discriminative information with reduced feature repetition.

#### 3.4.2 Wrapper based methods

Wrapper-based approaches choose the features as a subset when trained using a classifier as opposed to consideration of single feature. Optimization-based search algorithms were implemented and each candidate feature set was considered as a potential answer and assessed on the basis of the performance of the classifiers. In the current study, Ga and PsO techniques were used to select the contributing feature subset and respective hyperparameters of classifiers to analyze the cognitive activity.

##### 3.4.2.1 Genetic algorithm (Ga)

Ga (Munia et al., 2017) was used to determine a good combination of EEG features to analyze the cognitive activity. Instead of analyzing individual features, Ga uses a population of candidate feature subsets. The feature subsets are represented as binary strings (chromosomes) with each gene indicating a specific feature. Fitness of each chromosome is measured through the accuracy of the classifier and the population is updated on a successive generation. Ga parameters were set for the population size, genome length, crossover probability, mutation probability, elitism, tournament selection, and the maximum number of generations (Chiesa et al., 2020; Saibene and Gasparini, 2023).

Figure 7 shows, Ga based feature selection and hyperparameter tuning setup for cognitive analysis. The entire set of EEG features are fed to the Ga based optimization process. During optimization, the subset of features is chosen initially, and then the hyperparameters are fixed for a given classifier by iteratively selecting a particular feature subset. After all iterations, the subset of features yielded by the classifier that produced the highest accuracy was selected. These features are used to train and test the classifier.

**Figure 7:**
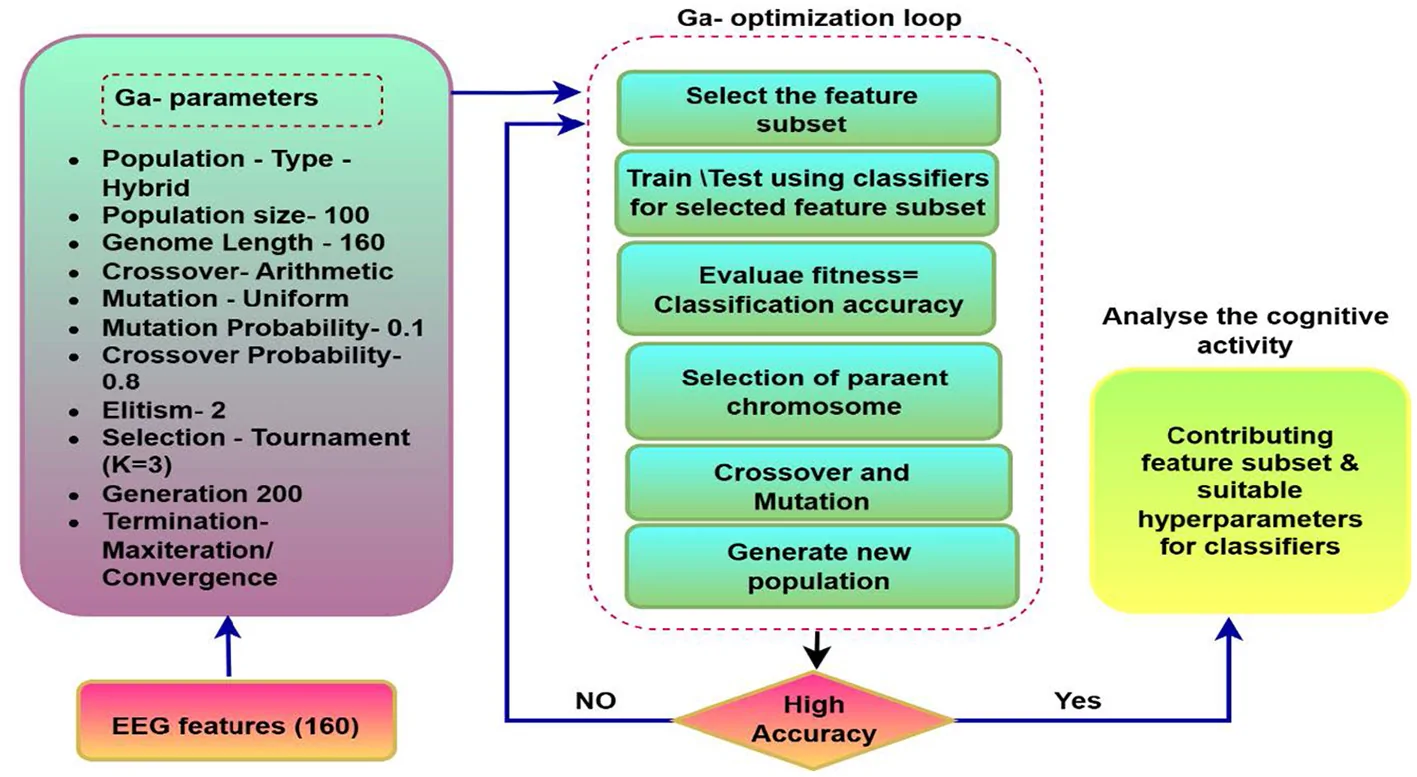
Ga-based feature selection and hyperparameter optimization workflow. A total of 160 EEG MEnt features is given as input to the Ga, where feature subsets and classifier hyperparameters are iteratively optimized using Ga parameters to obtain the best contributing feature subset with high classification accuracy.

##### 3.4.2.2 Particle swarm optimization (PsO)

PsO (Abualigah, 2025) was used to find the contributing feature subset and hyperparameters of classifiers to analyze the cognitive activity. Each instance of the solution in PsO is called a particle, a particle has a position vector that represents a subset of features and a velocity vector that controls change in the position. The classification accuracy of each feature subset is used to measure the efficacy of the particle. PsO was applied to improve feature selection for cognitive activity analysis through an iterative process by refining a contributing feature set, S. The entire set of EEG features were fed to the PsO algorithm. The initial PsO parameters were fixed for position length, iterations, inertia weight, cognitive coefficient, social coefficient, random factors and convergence conditions (Xu et al., 2023; Abualigah, 2025; Tran et al., 2019). Figure 8 depicts the selection of feature subset and suitable hyperparameters of classifiers.

**Figure 8:**
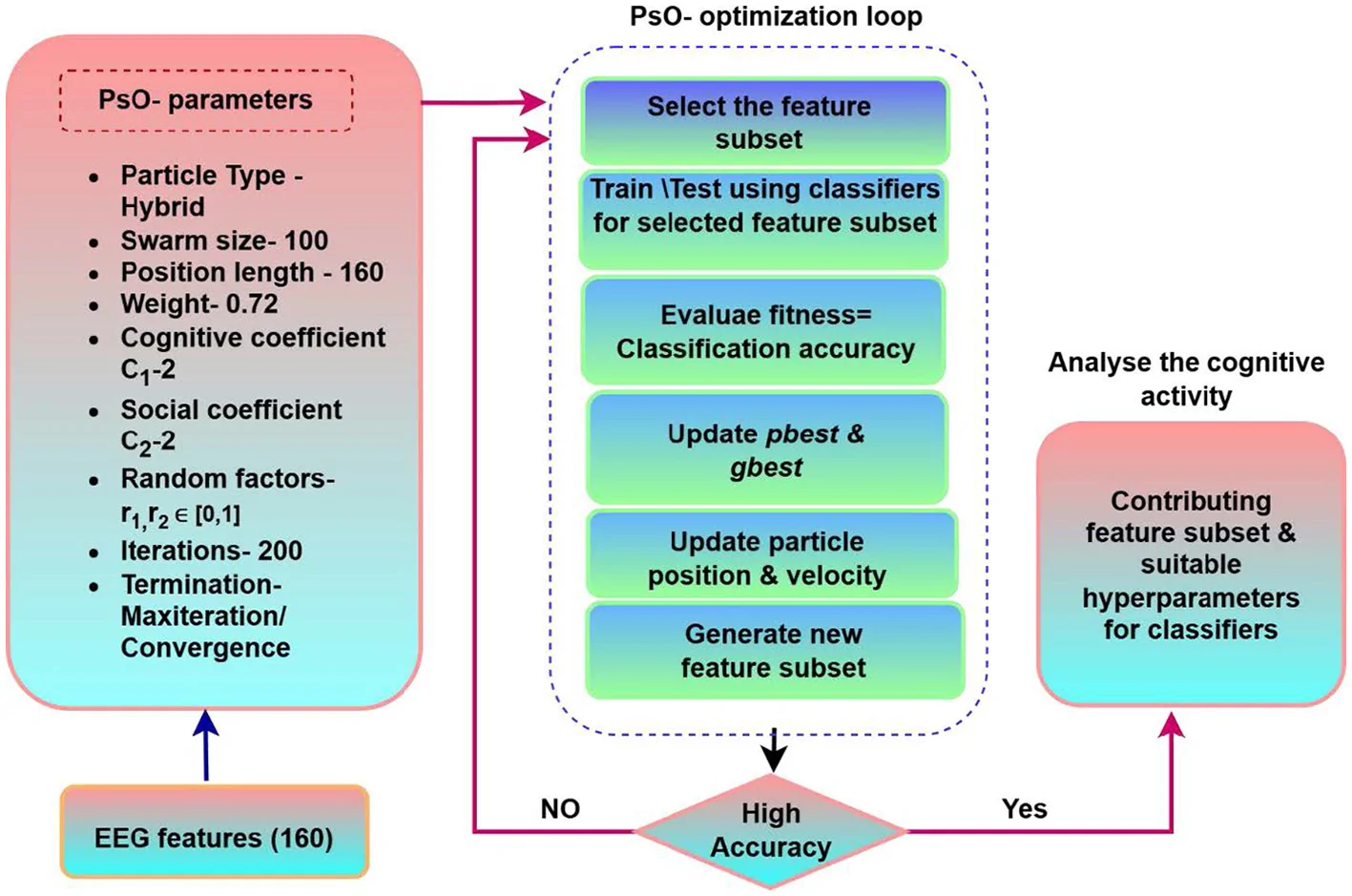
PsO-based optimization workflow for EEG feature selection. A total of 160 EEG MEnt features is given as input to the PsO. It updates particle position and velocity based on fitness evaluation to identify the most contributing feature subset and suitable classifier hyperparameters for achieving high classification accuracy.

### 3.5 ML classifiers

Supervised learning algorithms have wide application in the analysis of cognitive activity. In the present study five different ML (Tran et al., 2019; Probst et al., 2019a) classifiers (SVM, ANN, DT, KNN, RF) were used. In the phase-1 multiple classifiers were combined with metaheuristic optimization algorithms to find the more contributing feature subset. In the phase-2 these ML classifiers make use of the selected feature subset to classify the cognitive activity between sports and non-sports individuals.

### 3.6 Statistical analysis

Statistical tests were used to analyze the performance of the developed methodology. Welch's independent-samples *T*-test (West, 2021) was performed as given in the Equation 24. It was used to determine whether the selected EEG features differed significantly between the two groups. The feature-specific degrees of freedom were estimated using the Welch–Satterthwaite Equation 25. All tests were two-sided, and *p* < 0.05 was considered statistically significant.

$$\begin{array}{l} t\;=\;\frac{{\overset{-}{X}}_{1}-{\overset{-}{X}}_{2}}{\sqrt{\frac{s_{1}^{2}}{n_{1}}+\frac{s_{2}^{2}}{n_{2}}}}, \end{array}$$ (24)

$$v=\frac{{\left(\frac{s_{1}^{2}}{n_{1}}+\frac{s_{2}^{2}}{n_{2}}\right)}^{2}}{\frac{{\frac{(s_{1}^{2}}{n_{1})}}^{2}}{n_{1}-1}+\frac{{\frac{(s_{2}^{2}}{n_{2})}}^{2}}{n_{2}-1}}$$ (25)

*where*${\overset{-}{X}}_{1}$and ${\overset{-}{X}}_{2}-Mean\;feature\;values\;of\;two\;groups$$s_{1}^{2}$and $s_{2}^{2}-Corresponding\;variance$*n*_1_and *n*_2_−*Respective sample sizesv*−*degree of freedom*

Fleiss' kappa (Geijer et al., 2025) was subsequently employed to assess the overall consistency of the categorical decisions produced by the five ML classifiers. These classifiers were developed using the Ga-selected MEnt features obtained from the F3, F4, P3, and P4 channels. In the classification analysis 70:30 ratio was fixed. The five classifiers were considered raters, and their predicted labels (sports −1) and (non-sports −0) were treated as the two nominal categories. For participant *i*, agreement among the classifiers was calculated using Equation 26.

$$\begin{array}{l} P_{i}\;=\;\frac{1}{n\left(n-1\right)}\overset{k}{\underset{j\;=\;1}{\sum }}n_{ij}\left(n_{ij}-1\right) \end{array}$$ (26)

where*n* = 5 (number of classifiers)*k* = 2 categories of subjects*n*_*ij*_ (classifiers assigning participant *i*to category *j*)

$$\kappa \;=\;\frac{P-P_{e}}{1-P_{e}},$$ (27)

Fleiss' kappa was calculated using the Equation 27, where P represents the mean observed agreement and $P_{e}$ represents the agreement expected by chance. Kappa values were interpreted as slight (κ ≤ 0.20), fair (0.21–0.40), moderate (0.41–0.60), substantial (0.61–0.80), and almost perfect (0.81–1.00) agreement (Geijer et al., 2025). The *T*-test and Fleiss' kappa addressed different aspects of the analysis, the Welch's independent-samples *T*-test evaluated group differences in the EEG feature values, whereas Fleiss' kappa assessed the consistency of the sports/non-sports predictions across the five ML classifiers.

## 4 Results

The acquired EEG signals from the four electrode positions were subjected to preprocessing. ICA and bandpass filter were used to remove the unwanted frequency components and retain the frequency range between 8 and 30 Hz, respectively. This frequency was selected as it exhibits dominant alpha and beta rhythm activity associated with cognitive task-related neural activation. Figure 9 depicts the preprocessing analysis. The performance of the preprocessing method was assessed using the SNR, PSNR and processing time. SNR and PSNR quantified noise attenuation and signal fidelity, whereas processing time represented computational efficiency. Table 2 shows the analysis results of preprocessing technique.

**Figure 9:**
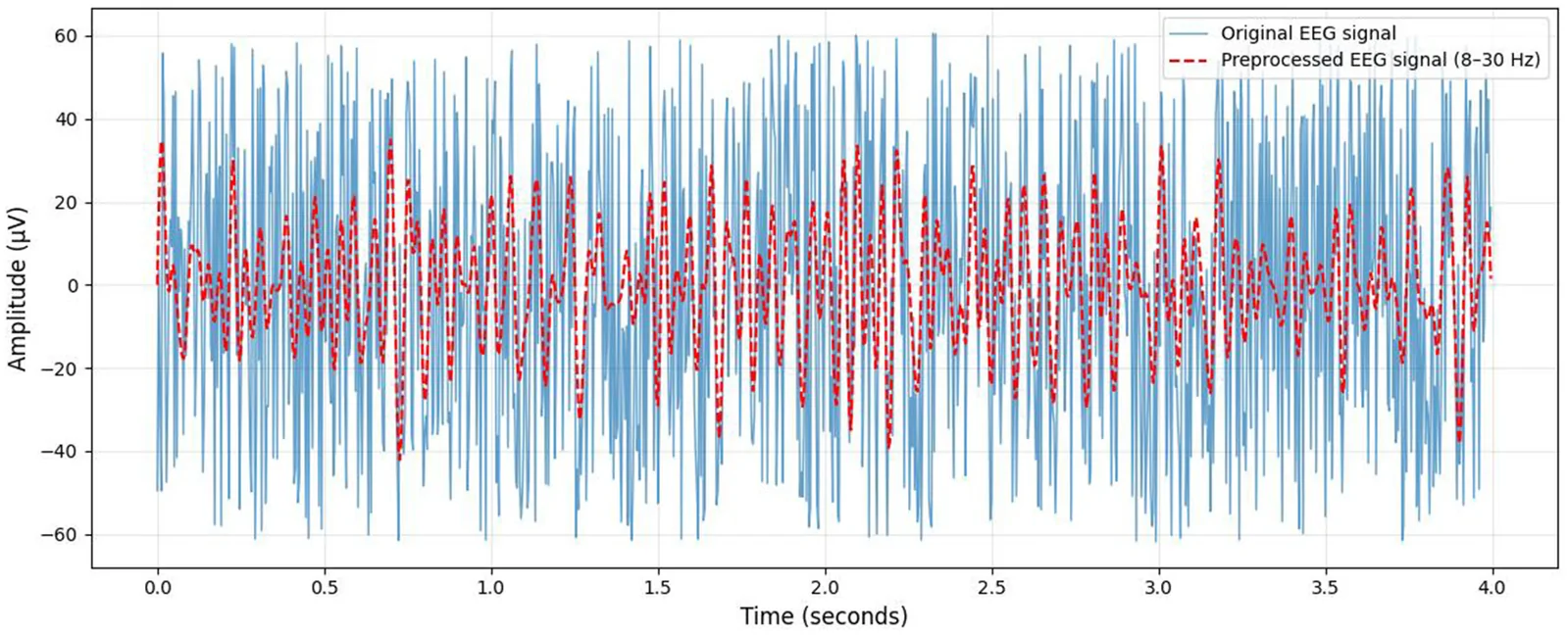
Signal preprocessing analysis. Comparison of the original EEG signal and the preprocessed EEG signal following ICA and third-order 8–30 Hz Butterworth band-pass filtering.

**Table 2:** Analysis of preprocessing technique.

S. no	Signal quality metrics (parameter)	Values
1	SNR (dB)	25.5011(dB)
2	PSNR (dB)	31.9212 (dB)
3	Processing time (seconds)	5.6295 (seconds)

SNR and PSNR were averaged across four 1-min EEG channels after filtering controlled 50-Hz interference; processing time indicates the total filtering duration.

In addition, signal integrity was assessed before and after preprocessing. The raw EEG recordings contained 13 missing samples, whereas no missing sample remained after preprocessing. No infinite values or clipped samples were detected at either stage. After preprocessing, 81.060 ± 5.153% of the signal power was retained within the 8 to 30 Hz range, indicating effective preservation of the frequency components relevant to the present analysis. From the preprocessed signal, entropy features were analyzed in terms of multiscale factors by using a method called coarse graining method. A signal-length sensitivity analysis was performed using the EEG recordings acquired in this study as presented in Table 3. The 15,360 samples correspond to 60 s of continuous EEG recorded at 256 Hz samples per channel ($N\;=\;F_{s}T\;=\;256^{*}60\;=\;15,360$). The sampling frequency provides a Nyquist frequency of 128 Hz, which is sufficient for the 8 Hz to 30 Hz EEG activity considered in the present analysis.

**Table 3:** Signal-length sensitivity analysis.

Duration (seconds)	Samples per channel	SaEnt	FzEnt	PrEnt	DpEnt
15 s	3,840	1.8289 ± 0.1525	1.6826 ± 0.1625	0.85999 ± 0.01839	0.91250 ± 0.03809
30 s	7,680	1.7963 ± 0.1383	1.6817 ± 0.1712	0.91034 ± 0.01632	0.91865 ± 0.03388
45 s	11,520	1.8039 ± 0.1170	1.6940 ± 0.1577	0.90088 ± 0.01528	0.92289 ± 0.03225
60 s	15,360	1.7910 ± 0.1419	1.6751 ± 0.1735	0.88177 ± 0.01527	0.92080 ± 0.03394

Signal-length sensitivity of entropy estimates at the P4 electrode and scale factor *t* = 10. Values are presented as participant mean ± SD.

However, the adequacy of the recording duration was evaluated separately through a within-dataset sensitivity analysis rather than inferred from the sampling frequency. For each participant and electrode position (F3, F4, P3 and P4), multiscale SaEnt, DpEnt, FzEnt and PrEnt were recalculated using cumulative recording durations of 15, 30, 45 and 60 s. These durations corresponded to 3,840, 7,680, 11,520, and 15,360 samples per channel, respectively. The same preprocessing procedure, entropy parameters and maximum scale factor of *t* = 10 was maintained for all signal lengths so that recording duration was the only factor varied. The entropy estimates became less sensitive to the addition of data at the longer recording duration. This sequence length was sufficient for applying the selected embedding parameters without producing undefined entropy values or numerical failures. Based on the analysis, the 60-s duration was retained and all the 4-entropy metrics were applied on the coarse-grained signal at four electrode positions (P3, F3, F4, and P4). In the present study, MDpEnt, MPrEnt, MSaEnt, and MFzEnt were calculated for the F3–A1, F4–A2, P3–A1, and P4–A2 derivations to characterize scale-dependent temporal complexity at the selected frontal and parietal locations. However, the features derived from these signals were subsequently integrated into a common feature vector for feature selection and classification. The scope of the present analysis is confined to the complexity characteristics represented by these derivations.

This resulted in a cumulative total of 160 MEnt features−4 (electrode positions) × 4 (entropy metrics) × 10 (scale factors). With the 160-feature set, it becomes difficult to identify which attributes truly contribute to group discrimination because many features are redundant, weakly informative, or correlated with each other. There is also the risk of overfitting because using all features may also contribute to a more complex model and make them less interpretable. To address this, both filter-based and wrapper-based selection strategies were implemented. MI was used to capture nonlinear dependency between each feature and the class labels, while Pearson correlation was applied to quantify linear association and remove highly correlated or less informative variables. Along with these methods the mRMR was used to retain EEG features that were strongly related to cognitive class discrimination while avoiding repeated or overlapping information from similar features. In addition, wrapper-based approaches such as Ga and PsO were adopted to search for a contributing feature subset. All MEnt features (*n* = 160) were first analyzed using MI to rank the features.

The same 160 MEnt features were then assessed using Pearson correlation as an additional filter criterion. Although Pearson correlation highlighted the same feature families (DpEnt, SaEnt, and FzEnt), the exact electrode scale combinations were not identical to those obtained from MI. This difference is expected because MI captures both linear and nonlinear dependence, whereas Pearson correlation quantifies only linear association. mRMR is another method that measures feature relevance with the class label similar to MI. However, it additionally considers redundancy among features during ranking. Hence, features with high relevance and low redundancy receive higher ranks. The top-ranked features were depicted in Figure 10. However, filter methods rank features individually and do not guarantee the best performing subset when features interact. Therefore, to identify a more contributing feature subset, search-based selection algorithms such as Ga and PsO were integrated with five different ML classifiers. Jointly optimization process encompassing both feature selection and hyperparameter tuning were accomplished as shown in Figures 7, 8. The performance of the Ga and PsO models were evaluated using nested cross-validation. The mean outer-fold accuracy and corresponding standard deviation (SD) obtained for each classifier are presented in Figure 11. The error bars represent the variation in accuracy across the outer test folds. Overall, Ga improved the mean classification accuracy by approximately 1–2 percentage points compared with PsO across the five classifiers. Ga also produced lower or comparable variability for most classifiers, particularly KNN, DT, and ANN.

**Figure 10:**
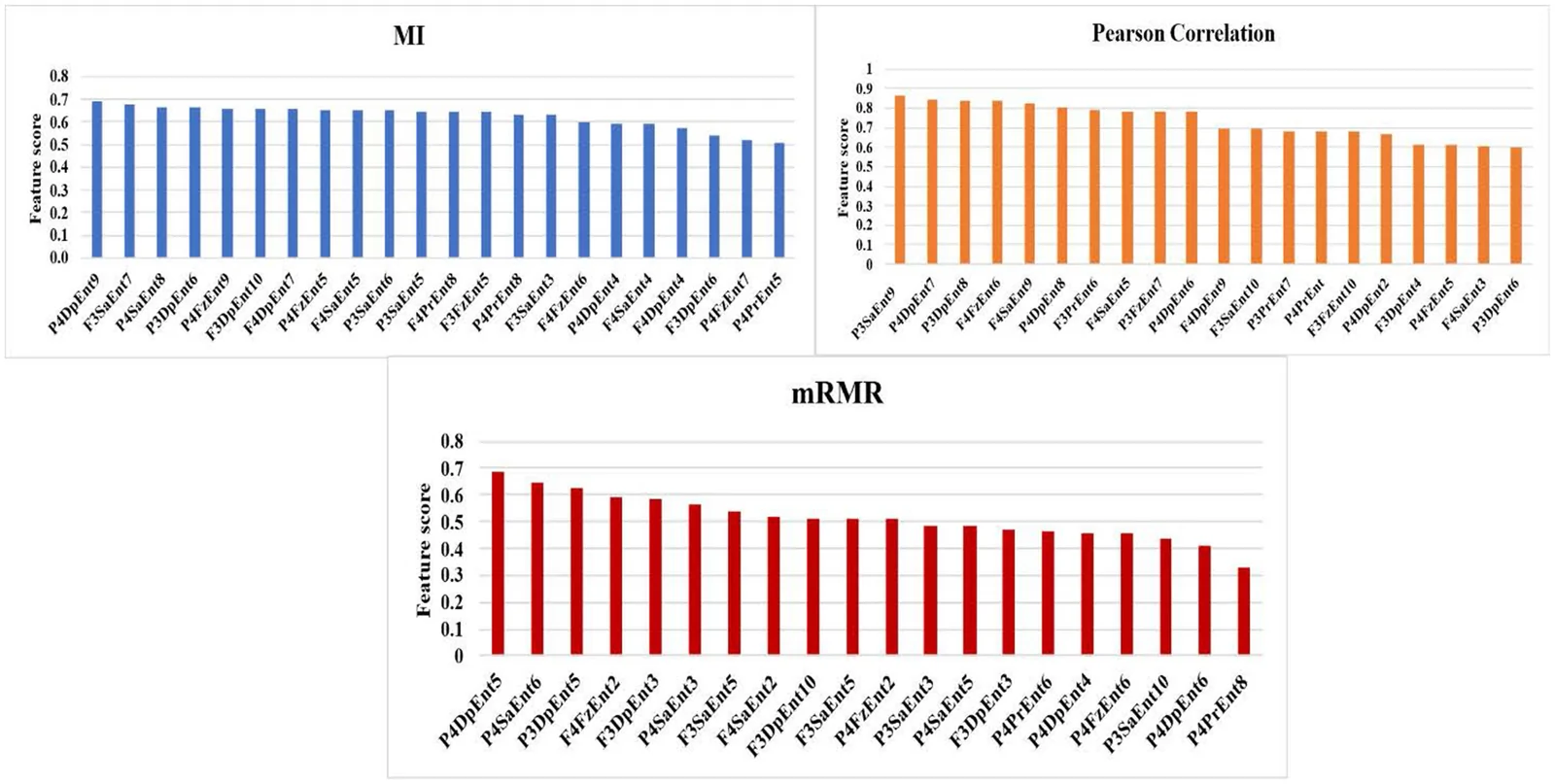
Filter-based ranking of MEnt features. MI, Pearson correlation, and mRMR rank the multiscale entropy features based on their relevance to cognitive activity analysis. The highest-ranked features indicate the most contributing electrode–entropy combinations.

**Figure 11:**
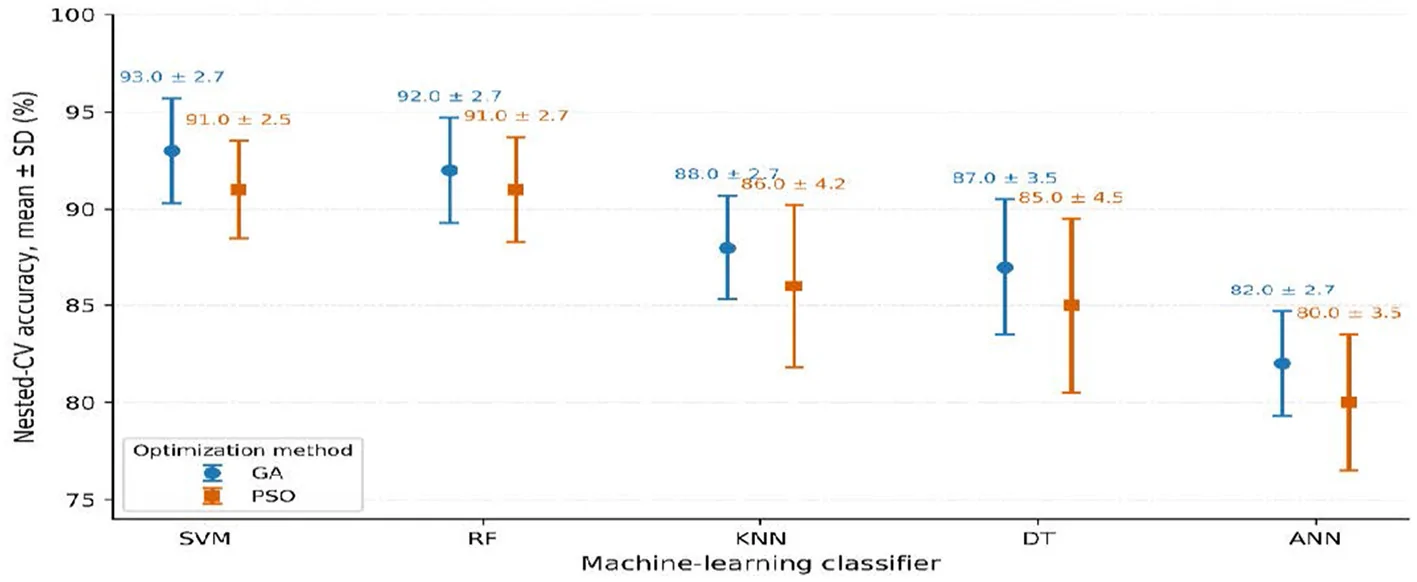
Comparative performance of Ga and PsO algorithms. The Ga and PsO algorithms are compared based on nested CV accuracy for SVM, KNN, ANN, DT, and RF models.

The feature-selection and hyperparameter-search procedures were performed separately for SVM, KNN, ANN, DT, and RF because the classifiers have different learning mechanisms and hyperparameter domains.

For every feature subset generated by the Ga, the candidate configurations within the predefined search spaces presented in Table 4 were evaluated using inner five-fold cross-validation. The configuration yielding the highest mean inner-fold accuracy was used to determine the fitness of the corresponding feature subset. During the search, the best inner-fold cross-validation accuracy was recorded at each generation. Figure 12 presents the convergence behavior of Ga–SVM across the five inner training folds. The accuracy increased during the initial generations and then remained nearly unchanged over the subsequent generations. No further improvement was observed before the maximum limit of 200 generations was reached, indicating convergence of the SVM search under the specified search bounds and stopping criterion. The same evaluation procedure and generation limit were applied independently to KNN, ANN, DT, and RF. This constitutes empirical evidence of convergence within the specified investigative framework and computational constraints. Since Ga is a stochastic metaheuristic, convergence in this study does not imply a mathematical guarantee of the global optimum. Rather, it indicates that the search had stabilized and no further improvement was obtained within the specified search space. Thus, each of the five procedures was considered to have reached a stable empirical convergence based on its fitness trajectory.

**Figure 12:**
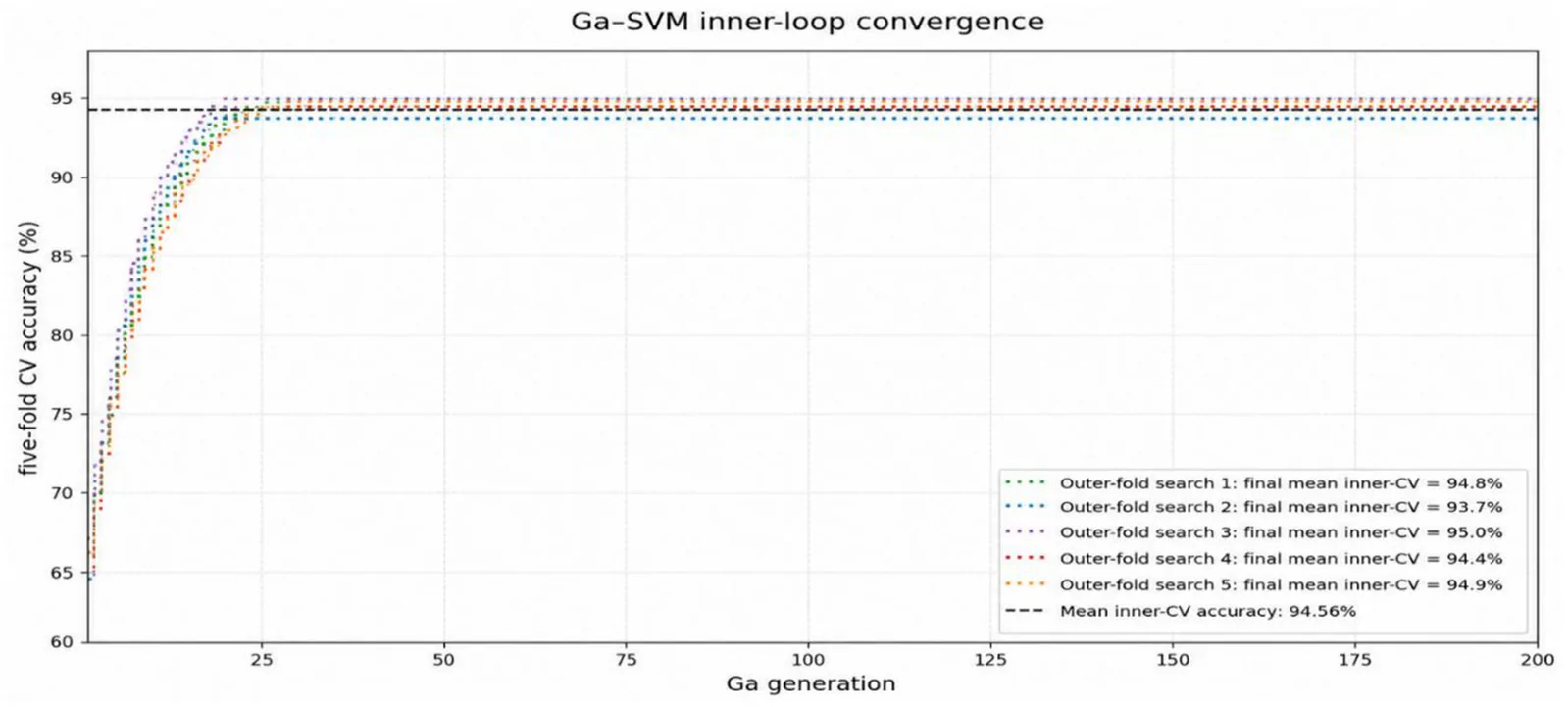
Ga-SVM optimization curve across generations. Fold-wise convergence behavior of the Ga–SVM framework under nested cross-validation for inner fold.

**Table 4:** Performance of the Ga-optimized classifiers under nested cross-validation.

Classifier and references	Hyperparameter search space	Selectedfeature count	Retained classifier configuration	Mean outer fold accuracy (%)	Wilson 95% CI
SVM (Probst et al., 2019a; Feurer and Hutter, 2020)	Kernel: RBF; *C* ε [0.1,100]; γ ε [0.001, 1.0]	13	Kernel—RBF *C*−1.35 Gamma−0.022	93.0 ± 2.7	83.6–96.6
KNN (Probst et al., 2019a)	*k* ε {3,…,15}; metric: Euclidean, Manhattan or Minkowski; weights: uniform or distance	19	*K*−4 Metric—Euclidean Weight—uniform	88.0 ± 2.7	80.2 – 93.0
DT (Probst et al., 2019a)	Criterion: Gini or entropy; max_depth ε[3,20]; min_samples_split ε[2,10]; min_samples_leaf ε[1,6]; ccp_alpha ε [0, 0.01]; class_weight: none or balanced	25	Max_depth−6 Min_samples_split−10 Min_samples_leaf−6 Ccp_alpha = 0.001 Criterion-gini Class_weight—balanced	87.0 ± 3.5	79.0–92.2
RF (Probst et al., 2019a; Bergstra and Bengio, 2012)	n_estimators ε [50, 500]; criterion: Gini or entropy; max_depth ε [3, 20]; max_features: sqrt or log2; min_samples_split ε [2, 10]; min_samples_leaf ε [1, 6]; ccp_alpha ε [0, 0.01]; class_weight: none or balanced	14	N_estimator−429 Max_depth−6 Min_samples_split−10 Min_samples_leaf−6 Ccp_alpha = 0.001 Criterion-gini Class_weight—balanced	92.0 ± 2.7	85.0–95.9
ANN (Probst et al., 2019a,b)	Activation function : ReLU or tanh; learning rate ε [0.001, 0.01]; schedule: constant or adaptive; momentum ε [0.8, 0.9]; batch size ε {10, 16, 32}; epochs ε {50, 100, 150}; solver: SGD	23	Activation function—ReLu Learning rate-−0.001 Learning rate schedule—adaptive Slover—SGD Momentum−0.9	82.0 ± 2.7	73.3–88.3

This table presents the hyperparameters selected for ML models and respective cross-validation accuracy.

Following completion of the inner search, the retained feature subset and classifier configuration from each outer training fold were evaluated once on the corresponding unseen outer test fold. As reported in Table 4, the mean outer-fold accuracies were 93.0 ± 2.7% for SVM, 88.0 ± 2.7% for KNN, 82.0 ± 2.7% for ANN, 87.0 ± 3.5% for DT and 92.0 ± 2.7% for RF. Table 4 also provides the predefined hyperparameter search spaces, selected-feature counts, retained classifier configurations and Wilson 95% confidence intervals. Thus, the retained configuration for each classifier was determined through inner cross-validation within explicitly defined search bounds and was subsequently evaluated using unseen outer-fold data. The reported configurations are therefore described as the best-performing solutions obtained within the specified search spaces and stopping criterion.

Ga–SVM provided the best balance between classification accuracy and feature-subset size. Since Ga–SVM achieved the highest nested cross-validation accuracy, its selected MEnt feature subset as shown in the Table 5 was further fed into the ML classifiers for analyse the cognitive activity between sports and non-sports participants.

**Table 5:** Ga-SVM selected feature subset.

Ga-SVM				
Entropy Metrics	DpEnt	SaEnt	FzEnt	PrEnt
MEnt Features	P4DpEnt7	P4SaEnt6	F4FzEnt6	P4PrEnt8
	P3DpEnt8	F3SaEnt8	F3FzEnt8	
	P4DpEnt5	F3SaEnt7	P4FzEnt5	
	P3DpEnt6	P3SaEnt3		
	F4DpEnt9			
Total	**5**	**4**	**3**	**1**
	**13**			

This table presents the Ga-SVM selected MEnt feature subset for cognitive activity analysis. The bold values indicate the total number of features selected in the subset.

The ML models were trained with a traintest split of 70:30. The performance metrics were analyzed using various metrics such as accuracy, sensitivity, precision, specificity and F1 score as shown in the Figure 13. These metrics depict the performance of all of the classifiers. In both groups SVM and RF classifiers performed well in discriminating the two groups with the highest classification accuracy of 96.67%. These findings are a strong indication that SVM and RF are especially suitable in cognitive activity analysis, which can be explained by the fact that these methods consistently yield reliable results provided that the study is conducted on different groups of population and under varying experimental conditions.

**Figure 13:**
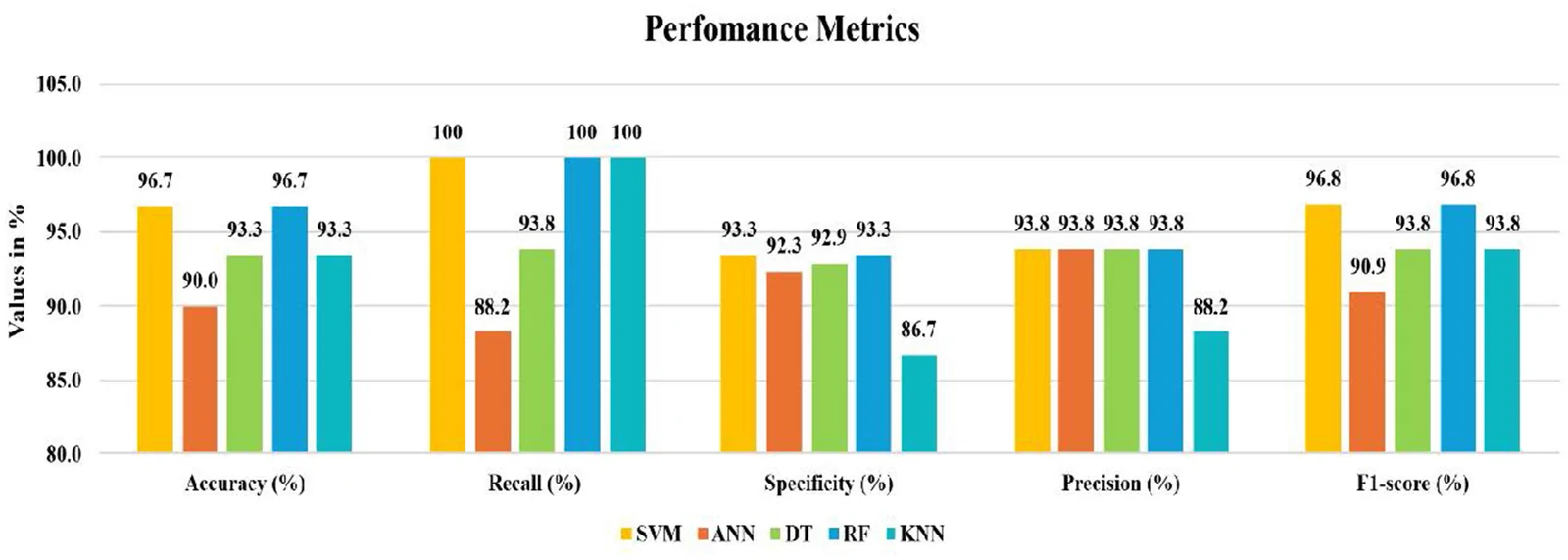
Classification performance for cognitive state analysis between sports and non-sports groups. All the ML classifiers are compared using accuracy, precision, recall, specificity, and F1-score, showing the performance variation.

Confusion matrices of the five classifiers are depicted in the Figures 14A–E. Class 0 corresponds to non-sports participants and class 1 corresponds to sports participants. The confusion matrices are a condensed representation of the model performance as they illustrate the percentage of correct and incorrect classifications of each of the classes. The majority of the misclassification was obtained in the non-sports samples while being classified as sports, which indicates that, there was overlap in the pattern of features between the two groups. This may be due to inter-individual variability as well as behavioral confounds that may have happened before recording including recent tasks that demand cognitive effort, intense exercise, yoga, meditation or any other activity that may involve attention and cognitive effort. The above activities may contribute to enhanced cerebral blood flow hence leaving the brain in an active condition leading to misclassification.

**Figure 14:**
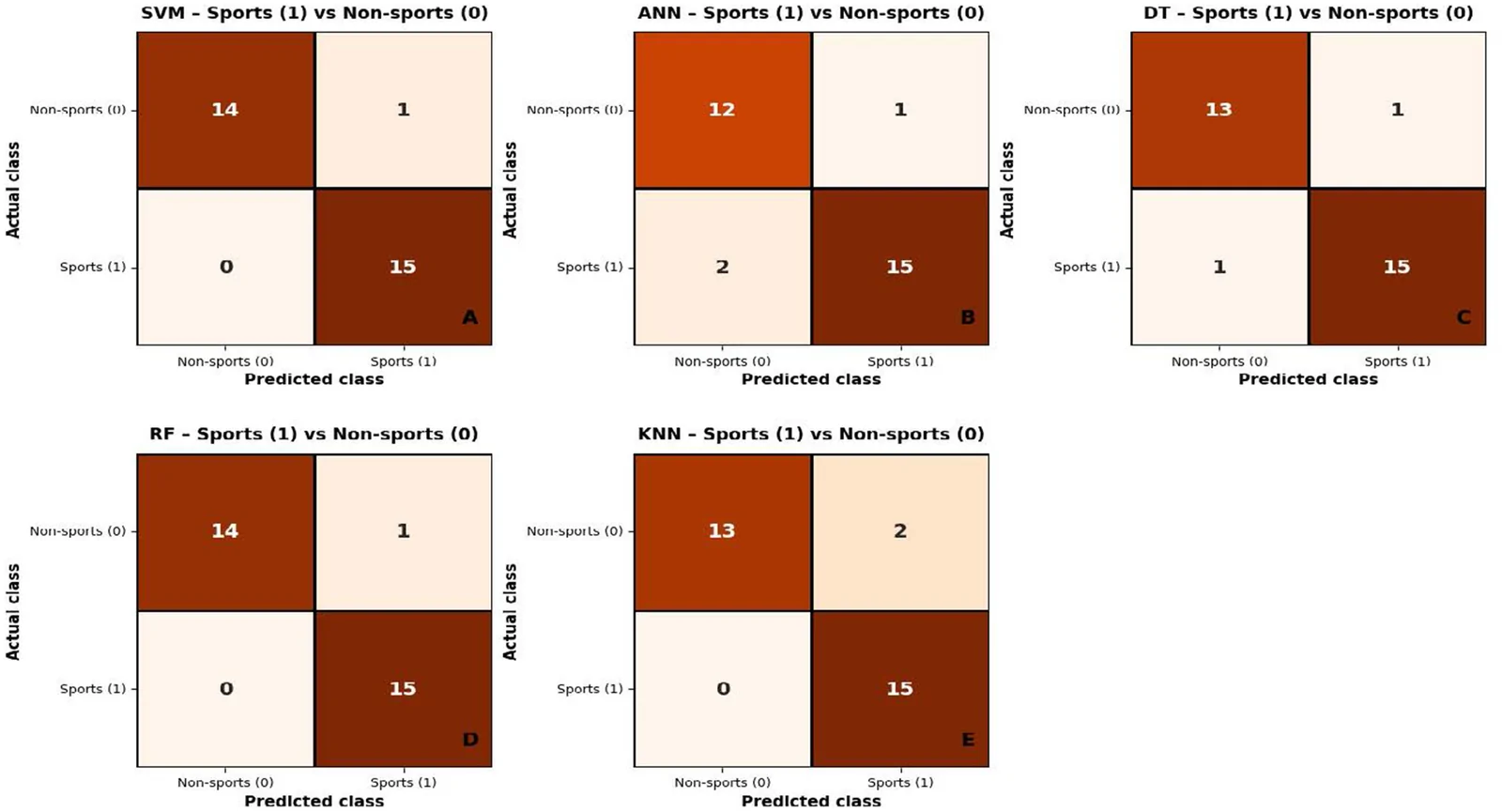
**(A–E)** Confusion matrices of ML classifiers for sports and non-sports classification. The confusion matrices show the classification outcomes of ML models.

Welch's independent-samples *T*-test was performed separately on all 160 MEnt features to evaluate differences between the sports and non-sports groups without assuming equal variances. At a two-sided significance level of *p* < 0.05, 117 features (73.1%) showed statistically significant group differences, whereas 43 features (26.9%) were not statistically significant. The feature-specific Welch–Satterthwaite degrees of freedom ranged from 49.71 to 98.00, reflecting feature-specific differences in group variances. Figure 15 depicts the box plots of representative MEnt features from the selected subset. The plots illustrate the median, interquartile range, overall spread and potential outliers within each group. Higher median values were observed in the sports group, and the comparatively limited overlap between the boxes indicate differences in the central tendency and distribution of the feature values between the two groups. The box plots therefore provide visual support for the group-level differences detected by Welch's test, while also showing within-group variability and the presence of outlying observations. The finding that 117 of the 160 MEnt features were statistically significant indicates that many EEG complexity measures had different mean values between the sports and non-sports groups. This suggests that the groups exhibited different multiscale EEG complexity patterns. *t*-value provides stronger evidence of a group difference. Its sign indicates which group has the higher mean. In case of degrees of freedom, values close to 98 indicate relatively similar variance contributions, whereas lower values indicate greater variance imbalance. Welch's adjustment makes the inference more reliable without imposing equal variance. In addition, Fleiss' kappa was used to evaluate the consistency of the sports/non-sports predictions produced by all classifiers. To eliminate the potential circularity, agreement was re-evaluated using a subject-wise stratified five-fold cross-validation procedure on the dataset. Fleiss' kappa was subsequently calculated from the predictions generated by the five classifiers for the held-out subjects. The mean fold-wise cross-validated Fleiss' kappa was therefore 0.874 ± 0.051, with a *t*-based 95% confidence interval of 0.811–0.937. All fold-wise values exceeded 0.80, indicating consistently high agreement among the classifiers on held-out subjects.

**Figure 15:**
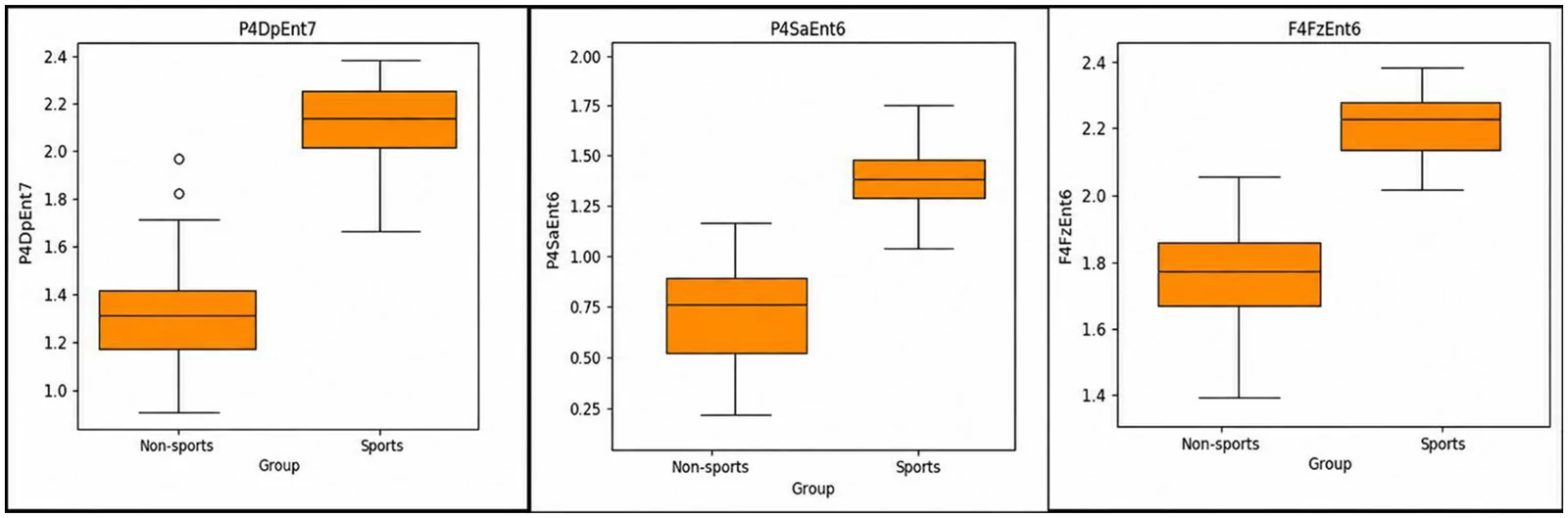
Box Plot for MEnt Features in the subset. The central line indicates the median, the box represents the interquartile range, the whiskers represent the non-outlier range, and the circles denote outliers.

## 5 Discussion

The proposed protocol distinguishes the feature wise differences using EEG among sports and non-sports individuals based on the recorded mental-arithmetic task. Patterns of neural activity differed among both the groups. The results indicated there is a difference in feature wise analysis among both groups. The reflecting differences in task-related cognitive activity are associated with attention, working memory, and mental processing rather than directly characterizing overall cognitive activity. Various studies have evaluated to analyze the impact of EEG using MEnt features in differentiating patterns of neural activity. Entropy features like, ApEnt, SaEnt, and PrEnt were primarily employed to analyze EEG signals related to cognitive, mild impairments and emotional states (Fernández-Valdés et al., 2023; Munia et al., 2017). These studies used single scale analysis. On the contrary, this study used MEnt features for analyzing cognitive activity of sports and non- sports individuals. The studies (Eroglu et al., 2020; Jung et al., 2000; Bergstra and Bengio, 2012) used MEnt features with less number of ML models to analyze the cognitive impairment, and various brain disorders. The statistical method like Fleiss kappa was not used for evaluating the inter-classifier agreement for classification of samples. In contrast this study utilizes five different classifiers and employs the Fleiss kappa statistical method to assess the inter-classifier agreement for classification of samples into sports and non-sports individuals.

### 5.1 Independent methodological evaluation using the Physiobank dataset

A set of experiments were performed with the help of a publicly available Physiobank dataset (Chen et al., 2023) to independently evaluate the proposed electrode-selection and EEG analysis methodology under cognitive-state variation. Various studies have used the Physiobank dataset for cognitive related studies (Iinuma et al., 2022; Goldberger et al., 2000; Saini et al., 2020; Zyma et al., 2019). This dataset has two classes which includes active and resting states recorded from healthy participants aged between 19 and 25 years. EEGs were captured for 1 min using a set of 12 scalp electrodes on a Neurocom 23-channel system. EEG signals from P3, P4, F3, F4 electrode positions and the same preprocessing strategy comprising ICA with bandpass was 8implemented. Four different entropy metrics PrEnt, DpEnt, SaEnt, and FzEnt, were applied in the coarse-grained method for four EEG electrode locations P3, P4, F3, and F4. MEnt features were extracted in the scale range of (*t* = 1 to *t* = 10), which resulted in 160 MEnt features. Ga based optimization was used to select the most discriminative feature subset and suitable hyperparameters for ML classifiers.

Table 6 lists the feature subsets selected by Ga-SVM for the benchmark dataset. Figure 16 shows the results of Ga based ML classification on the Physiobank dataset depicting the accuracy and contributing feature subset. Therefore, the Physiobank experiment should not be interpreted as an external validation of the sports-vs.-non-sports classification model. Instead, it provides an independent methodological evaluation of the proposed electrode-selection, MEnt feature-extraction, and classification procedure under cognitive-state variation. In both datasets, MEnt features were used to characterize EEG patterns associated with cognitive activity. The findings indicate that the proposed methodology can differentiate cognitive patterns in an independent EEG dataset. However, the proposed framework may be robust and transferable, and it is capable of capturing underlying neural processes.

**Table 6:** Analysis of Physiobank dataset.

Physiobank data (Ga-SVM)				
Entropy Metrics	DpEnt	SaEnt	FzEnt	PrEnt
MEnt features	P3DpEnt10	P3SaEnt8	P4FzEnt8	P4PrEnt8
	P4DpEnt8	F3SaEnt9	P3FzEnt7	P3PrEnt6
	P4DpEnt5	P4SaEnt7		
	P3DpEnt7	P4SaEnt6		
	F4DpEnt6	F4SaEnt8		
	F3DpEnt9	P4SaEnt5		
	P3DpEnt5			
	F3DpEnt5			
Total	**8**	**5**	**2**	**2**
	**17**			

This table presents the Ga-SVM selected MEnt feature subset for cognitive activity analysis in the Physiobank dataset. Combination of features were selected for Ga-SVM. The bold values indicate the total number of features selected in the subset.

**Figure 16:**
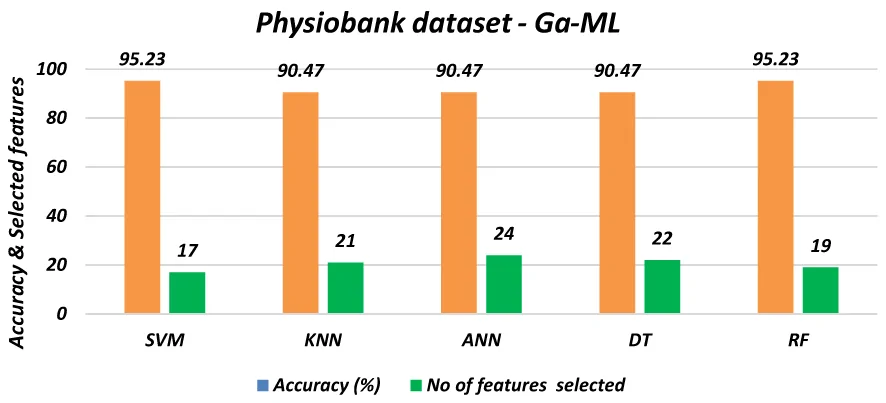
Ga-ML classification performance on the Physiobank dataset. The Ga-optimized ML classifiers are compared based on classification accuracy and contributing features.

## 6 Conclusion

The current study evaluvated the impact of MEnt features in analyzing the cognitive activity between sports and non-sports individuals. The entropy-based features were extracted from the frontal and parietal electrodes that captured meaningful changes in signal complexity across multiple scales. The feature selection stage adopted both filter and wrapper-based methods to select the MEnt features that contributed strongly to cognitive analysis, which reduced redundancy and improved the practical usefulness of the model. Among the various methods, Ga outperformed by selecting smaller and more effective feature subset. The same feature subset was used for differentiating the cognitive activity between sports and non-sports individuals with SVM and RF each reaching the higher classification accuracy with fewer misclassifications than the other models. The proposed MEnt feature-extraction and classification procedure was independently evaluated under cognitive-state variation using the Physiobank dataset. These findings support that MEnt features, when selected with proper feature selection strategies serve as a valuable metric for cognitive activity analysis. The study also suggests that such an approach can be useful in sports research for examining cognitive differences and training related evaluation. Future analysis will employ multivariate multiscale entropy and functional connectivity measures for the direct characterization of mutual temporal dependencies and coordinated frontal–parietal dynamics. Gender wise comparison, and combined evaluation of fitness traits with EEG derived cognitive measures can also be analyzed.

## Data Availability

Requests for data access should be addressed to the corresponding author.
